# Odorant Receptors Mediating Avoidance of Toxic Mustard Oils in *Drosophila melanogaster* Are Expanded in Herbivorous Relatives

**DOI:** 10.1093/molbev/msaf164

**Published:** 2025-07-04

**Authors:** Teruyuki Matsunaga, Carolina E Reisenman, Benjamin Goldman-Huertas, Srivarsha Rajshekar, Hiromu C Suzuki, David Tadres, Joshua Wong, Matthieu Louis, Santiago R Ramírez, Noah K Whiteman

**Affiliations:** Department of Complexity Science and Engineering, Graduate School of Frontier Sciences, The University of Tokyo, Chiba, Japan; Department of Molecular and Cell Biology, University of California Berkeley, Berkeley, CA, USA; Department of Integrative Biology, University of California Berkeley, Berkeley, CA, USA; Department of Molecular and Cell Biology, University of California Berkeley, Berkeley, CA, USA; Department of Molecular and Cell Biology, University of California Berkeley, Berkeley, CA, USA; Department of Molecular, Cellular, and Developmental Biology, University of California Santa Barbara, Santa Barbara, CA, USA; Department of Pathology, Johns Hopkins University School of Medicine, Baltimore, MD, USA; Department of Molecular, Cellular, and Developmental Biology, University of California Santa Barbara, Santa Barbara, CA, USA; Department of Evolution and Ecology, University of California Davis, Davis, CA, USA; Department of Molecular and Cell Biology, University of California Berkeley, Berkeley, CA, USA; Department of Integrative Biology, University of California Berkeley, Berkeley, CA, USA

**Keywords:** *Drosophila melanogaster*, olfaction, isothiocyanate, odorant receptor, Or42a, *Scaptomyza flava*, herbivory, evolution, Brassicales, mustard plants, AlphaFold2

## Abstract

Plants release defense volatile compounds that can deter herbivores. Among them are electrophilic toxins, such as isothiocyanates from mustard plants, that activate pain receptors by contact (i.e. taste) in many animals, including *Drosophila melanogaster*. While specialist insects have evolved strategies to tolerate toxicity and use mustard plants as hosts, it is unclear whether nonspecialist insects detect and avoid electrophilic toxins via olfaction. To address this, and to understand if specialized insects co-opted these toxic compounds as host plant olfactory cues, we leveraged closely related drosophilid species, including the microbe-feeding *D. melanogaster* and *Scaptomyza pallida*, and the mustard-feeding specialist *Scaptomyza flava*. In olfactory assays, *D. melanogaster* exposed to allyl isothiocyanate volatiles were rapidly immobilized, demonstrating the high toxicity of this wasabi-derived compound to nonspecialists. Through single sensillum electrophysiological recordings from olfactory organs and behavioral assays, we identified an olfactory receptor (Or) necessary for volatile detection and behavioral aversion to allyl isothiocyanate in *D. melanogaster*. RNA-sequencing and heterologous expression revealed that *S. flava* possess lineage-specific, triplicated homologs of this *Or* and that each paralog exhibited broadened and distinct sensitivity to isothiocyanate compounds. Using AlphaFold2 modeling, site-directed mutagenesis, and electrophysiological recordings, we identified two critical amino acid substitutions that changed the sensitivity of these paralogs from fruit-derived odors to isothiocyanates in the mustard specialist *S. flava*. Our findings show that nonspecialists can detect electrophiles via olfaction and that their olfactory systems can rapidly adapt to toxic host plant niches through co-option and duplication of ancestral chemosensory genes with few amino acid changes.

## Introduction

Plants have evolved the ability to synthesize a diverse array of toxic specialized metabolites that can provide resistance against insect herbivory. In turn, herbivorous insects have evolved diverse morphological, physiological, and behavioral counter-strategies to avoid these chemicals if encountered, or to mitigate their effects if ingested (Mithöfer and Boland 2012). Some herbivores even co-opt these plant toxins as oviposition or feeding stimulants (and even as chemical defenses of their own). For example, monarch butterflies evolved insensitivity against cardenolides released from their milkweed host plants (Reichstein et al. 1968; Dobler et al. 2012). Many plant toxins are, however, far more promiscuous in their modes of action, which presents a different “evolutionary hurdle” (Southwood 1972) to herbivores. Among them are various alkaloids, terpenoids, and electrophilic green leaf volatiles (Noge and Becerra 2015; Yaffe et al. 2015; Iorio et al. 2022) that intoxicate and deter herbivores by forming covalent bonds with biological molecules (War et al. 2012).

Mustard plants (Brassicales: Brassicaceae) such as thale cress (*Arabidopsis thaliana*), arugula (*Eruca sativa*), and wasabi (*Eutrema japonicum*) have evolved a sophisticated chemical defense system that produces electrophilic toxins upon wounding (Ahuja et al. 2010). These plants produce nontoxic glucosinolates, some of which are hydrolyzed *in planta* to form toxic electrophilic compounds such as isothiocyanates (ITCs) (Hopkins et al. 2009). ITCs are reactive compounds defined by a −N = C = S functional group attached to an R group, wherein the electron-deficient carbon is attacked by nucleophiles. Examples of ITCs include allyl ITC (AITC) derived from wasabi and radish *Raphanus sativus* (Cuellar-Nuñez et al. 2022) and butyl ITC (BITC) derived from the cabbage *Brassica oleracea* (MacLeod et al. 1989). The chemical diversity of glucosinolates allows Brassicales plants to effectively deter a wide array of herbivorous insects because different species have different mixtures of glucosinolates, making it more difficult for insects to adapt (Winde and Wittstock 2011).

Leaf-mining drosophilid flies in the genus *Scaptomyza* (e.g. *Scaptomyza flava* and *Scaptomyza montana*) have evolved to cope with these toxic Brassicales metabolites and are obligate herbivores phylogenetically nested within the paraphyletic *Drosophila* subgenus. These *Scaptomyza* mustard specialists, through rapid gene duplication and nonsynonymous changes, have evolved some of the most efficient ITC-detoxifying enzymes known from animals (Gloss et al. 2014, 2019).

While detoxification mechanisms help animals cope with at least some noxious compounds, sensory detection and behavioral avoidance of these substances can act as a checkpoint to prevent intoxication. Indeed, insects can behaviorally avoid toxic chemicals through gustation and/or olfaction (Bernays and Chapman 1987; Stensmyr et al. 2012; Scott 2018; Chen and Dahanukar 2020; Dweck and Carlson 2020). This includes avoidance of ITCs: exposure to volatile mustard plant extracts kills *Drosophila melanogaster* (Lichtenstein et al. 1964), and physical contact with AITC triggers repulsion via gustatory receptor cells that express the nociceptive “wasabi receptor” TrpA1 and Painless (Al-Anzi et al. 2006; Kang et al. 2010; Kim et al. 2010; Mandel et al. 2018). Additionally, volatile AITC causes behavioral aversion in fire ants (*Solenopsis invicta*) (Hashimoto et al. 2019). However, the functional and genetic basis underlying olfactory detection and avoidance of electrophilic toxins like ITC remain poorly understood.

*Scaptomyza* species include both nonherbivorous (e.g. microbe-feeding) and herbivorous species that use Brassicales plants (Aguilar et al. 2024). Herbivorous species have lost olfactory receptors that ancestral microbe-feeding species (Whiteman et al. 2011, 2012; Peláez et al. 2023) like *D. melanogaster* use to detect fermentation, microbial, and fruit odors (Goldman-Huertas et al. 2015). Likely to aid host plant location, the herbivorous specialist *S. flava* has evolved paralogous copies of the Olfactory receptor 67b (which is expressed in antennal olfactory sensory neurons, OSNs) that respond to ITCs. In contrast, the single copy of microbe-feeding *D. melanogaster* and *Scaptomyza pallida* respond to green leaf volatiles like trans-3-hexenol but not to ITCs (Matsunaga et al. 2022). Although these findings provide insight into how sensory receptors evolved in specialists, how evolutionary changes facilitate aversion, and/or attraction to toxic compounds is still largely unknown, yet it is a central problem in understanding how organisms invade toxic niches and herbivorous insects specialize on toxic host plants.

**Fig. 1.**
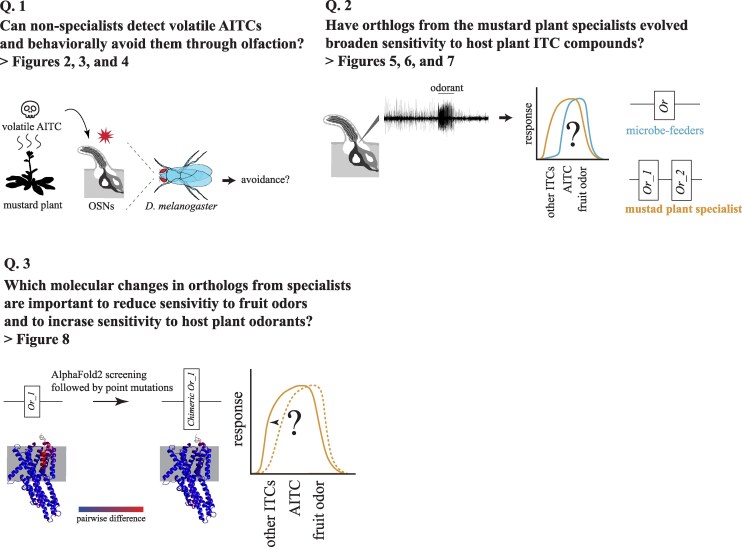
Questions addressed in this study.

In this study, we addressed the following three questions (Fig. 1): (1) Do nonspecialist insects detect toxic volatile ITCs via olfaction and behaviorally avoid them? (2) Have the homolog olfactory receptors of specialist insects evolved broader sensitivity to ITC host plant compounds? (3) If so, what molecular changes in these homologous olfactory receptors underlie this broadened sensitivity? To address these questions, we first conducted behavioral toxicity assays and found that *D. melanogaster* is rapidly immobilized by volatile exposure to AITC. We demonstrated that olfactory detection and behavioral avoidance of this compound requires Olfactory receptor 42a (Or42a), which is expressed specifically in maxillary palp OSNs (Ray et al. 2007). Next, in the specialist species *S. flava*, we discovered that the *Or42a* paralog is triplicated and that the number of Or42a-positive OSNs is expanded. Concomitantly, we found that these paralogous Or42a proteins are collectively sensitive to a broad range of ITC compounds, whereas the single Or42a copies encoded in the genomes of the microbe-feeding *S. pallida* and *D. melanogaster* respond only to AITC. Finally, AlphaFold2 3D modeling, site-directed mutagenesis, and electrophysiological experiments identified two key amino acid replacements that shifted the sensitivity of *S. flava* Or42a paralogs from fruit odors to ITCs. Collectively, our findings demonstrate that plant-derived volatile toxins like ITCs negatively impact nonspecialists and are detected via olfaction to mediate avoidance, and that gene duplication events and tuning shifts of olfactory receptors are coupled with specialization of herbivorous insects onto toxic host plants.

## Results

### Volatile AITC Rapidly Immobilizes *D. melanogaster*

**Fig. 2.**
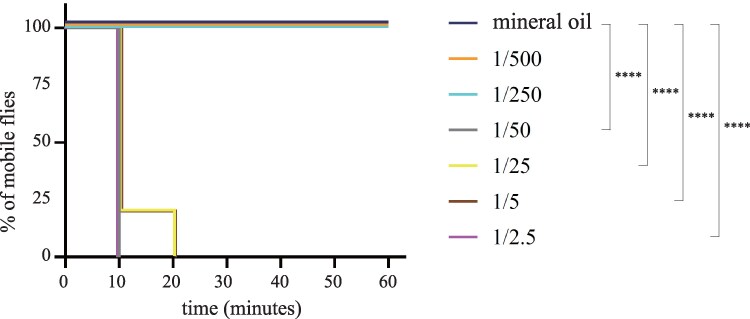
*Drosophila melanogaster* is immobilized rapidly upon exposure to volatile AITC. The toxic effect of various concentrations (vol/vol) of volatile AITC to *D. melanogaster* was assessed by measuring the % of mobile flies; flies were not allowed to contact the AITC source (supplementary fig. S1A, Supplementary Material online). Increasing the concentration of AITC decreases the % of mobile flies, likely due to intoxication. *n* = 10 for each condition (solvent and concentration). ****P < 0.0001, log-rank Mantel–Cox tests against the control.

To assess the toxic effects of volatile AITC in *D. melanogaster*, we conducted immobility assays with various concentrations of volatile AITC (from 1:500 to 1:2.5 vol/vol). In our experimental setup, a fabric mesh separated the chamber containing the flies from the chamber containing the AITC solution, and therefore, flies were exposed to volatile AITC but could not contact (i.e. taste) the AITC solution directly (supplementary fig. S1a, Supplementary Material online). While all insects in both the control treatment and those exposed to AITC 1:250 and 1:500 vol/vol remained active, most flies exposed to AITC concentrations ≥1:50 vol/vol became paralyzed (likely highly intoxicated or even dead) within 10 min (Fig. 2). Thus, this rapid immobilization indicates that plant-derived electrophilic compounds like AITC can have a strong negative effect on flies, consistent with previous findings (Lichtenstein et al. 1964).

### Volatile AITC Is Detected by the Maxillary Palp Olfactory Receptor Or42a in *D. melanogaster*

**Fig. 3.**
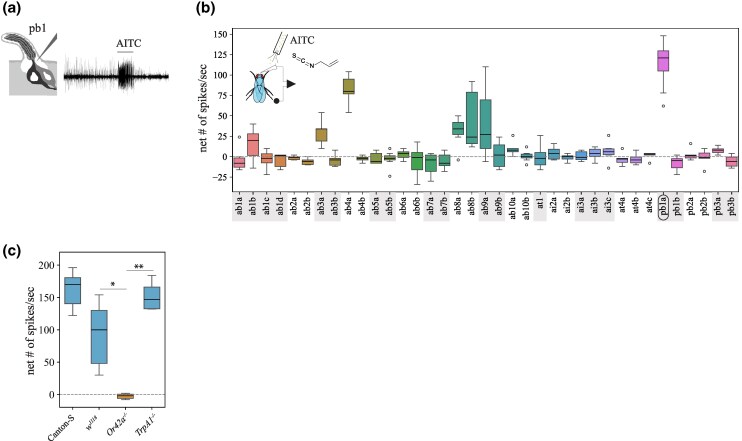
*Drosophila melanogaster* Or42a mediates detection of volatile AITC. a) Schematic and representative trace of an SSR from pb1 OSNs upon stimulation with AITC 1:100 vol/vol. The horizontal bar indicates the onset of the stimulus and its duration (1 s). b) SSR from all *D. melanogaster* antennae and palp basiconic sensilla upon AITC stimulation (1:100 vol/vol; *n* = 6 to 10 recordings/sensilla type from six animals). Represented here and in all figures are the control-subtracted net number of spikes/s, unless otherwise noted. The horizontal dotted line at zero indicates no response to odor stimulation. Here and thereafter, horizontal bars represent the median, the edges of the boxes correspond to 25th and 75th quartiles, the whiskers denote 10th and 90th quartiles, and symbols indicate outliers. Pb1a sensilla, which house Or42a (Ray et al. 2007), respond strongly to AITC. c) Responses from pb1 sensilla of wild-type Canton-S, the genetic background control *w^1118^*, *Or42a^−/−^*, and *TrpA1^1^ D. melanogaster* flies to 1:100 vol/vol AITC stimulation (*n* = 6 to 10 sensilla/genotype from three to four animals/genotype). The responses of both mutant flies were compared against each other and against those of *w^1118^*. Kruskal–Wallis ANOVA followed by Dunn's multiple comparisons: *P < 0.05; **P < 0.01.

The high toxicity caused by volatile AITC indicates that this volatile had the potential to be detected by the fly's olfactory system and that this could provide a fitness benefit if these volatile toxins were then behaviorally avoided. We investigated this in *D. melanogaster* by conducting exhaustive single sensillum recordings (SSRs) from the fly's olfactory organs, the antennae, and the maxillary palps, upon stimulation with volatile AITC. Several OSNs showed excitatory responses to AITC, but OSNs in palp basiconic sensilla 1a (pb1a) were the most activated (Fig. 3a and b, >100 spikes/s). Because pb1a OSNs express Or42a (Couto et al. 2005), we investigated whether OSN responses in this sensilla type are indeed mediated by this olfactory receptor. OSNs in pb1 sensilla from genetic background control flies (*w^1118^*) showed strong responses to volatile AITC, while these OSNs showed no response in *Or42a*^−/−^ flies (Fig. 3c, <2 spikes/s), indicating that Or42a detects volatile AITC in these OSNs. Furthermore, OSNs in pb1a of *TrpA1^1^* null mutant flies responded strongly to AITC (>150 spikes/s, Fig. 3c), showing that the contact chemoreceptor TrpA1 is not necessary for maxillary palp olfactory detection of this compound.

### Volatile AITC Repels *D. Melanogaster* via the Olfactory Receptor Or42a

**Fig. 4.**
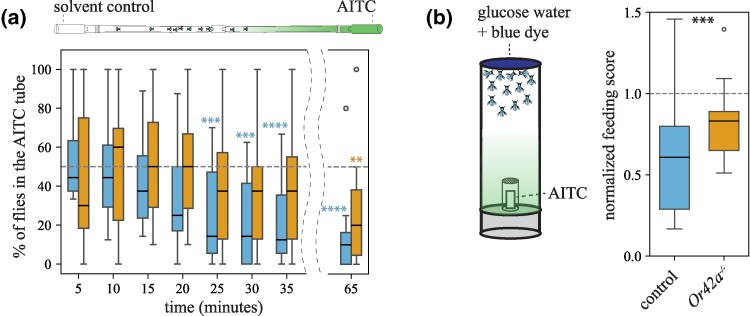
*Drosophila melanogaster* Or42a mediates behavioral aversion to AITC. a) Positional olfactory assay. Flies could smell, but not contact, the AITC solution (supplementary fig. S1B, Supplementary Material online). The number of flies in the odorless and the odorous glass tubes was counted every 5 min until 35 min, and then again at 65 min. The dotted line at 50% indicates random distribution between the two tubes. Genetic background control flies (line # 68384, blue boxes, *n* = 15) avoided the tube closest to the odor source at various time points (***P < 0.005; ****P < 0.001; one-sample signed rank tests against median = 50%). *Or42a^−/−^* mutants (orange boxes, *n* = 15) distributed randomly between the two tubes at all time points (*P* > 0.05) except at 65 min (*P < 0.01). b) Food consumption assay in presence or absence of AITC volatiles (1:500 vol/vol). Flies could smell but not contact the AITC solution (supplementary fig. S1C, Supplementary Material online). Both genetic background control flies (*n* = 16) and *Or42a^−/−^* mutants (*n* = 14) fed less in the presence of AITC volatiles (median < 50%, one-sample signed rank tests on normalized data; *P* < 0.05 and *P* < 0.001, respectively), but the feeding score of genetic control flies was lower than that of mutant flies (Mann–Whitney *U* test, ***P < 0.001). The dotted line at 50% indicates no feeding aversion or enhancement.

Next, we examined if Or42a plays a role at the behavioral level. We conducted a positional olfactory assay based on Ohashi and Sakai (2015) with modifications to prevent flies from physically contacting the odor source. Female flies (*n* = 10 to 12) were released in a dispositive consisting of two glass tubes, each of which was connected to a vial containing an odor solution or a vial loaded with a solvent (supplementary fig. S1b, Supplementary Material online). The number of flies in each tube (hereafter “odorless/control tube” and “odorous/test tube”) as well as in the release section was counted every 5 min up to 35 min (and again at 65 min). To validate this assay, we confirmed that wild-type *D. melanogaster* flies (strain Canton-S) showed normal olfactory-guided behavior in this behavioral setup using apple cider vinegar (one-sample signed rank tests, *P* < 0.05 in all cases, *n* = 21, supplementary file 4, Supplementary Material online), a well-established *D. melanogaster* olfactory attractant (Semmelhack and Wang 2009; Becher et al. 2010). We then tested whether AITC causes olfactory repellence. For these and all forthcoming behavioral experiments, we used as a genetic background control an “empty Gal4 control” line (line # 68384) which carries *white* in the background of a *white* mutation, as in all the mutant fly lines. These control flies and wild-type Canton-S flies avoided the AITC tube at various time points (25, 30, 35, and 65 min, Fig. 4a and supplementary fig. S2a, Supplementary Material online). In contrast, *Or42a^−/−^* mutants avoided the AITC tube only at the 65 min time point (Fig. 4a), possibly by taste detection of AITC via TrpA1 (volatile AITC molecules might have adhered to the glass tube walls at this point). Importantly, we confirmed that *Or42a^−/−^* mutants are capable of odor-mediated olfactory orientation in dual-choice trap assays offering apple cider vinegar versus water (described in Matsunaga et al. 2022): all captured flies were recruited to the odor-baited trap (*N* = 8 tests, *n* = 20 females/test; Wilcoxon-matched pairs test, *P* < 0.005; supplementary file 4, Supplementary Material online). These results suggest that *Or42a* plays a crucial role in mediating olfactory-driven behavioral aversion to AITC.

Given that detection of food-related volatiles is known to increase sugar consumption (Reisenman and Scott 2019) and that palp OSNs are located near the mouthparts, we reasoned that volatile detection of toxic/aversive odors such as AITC would instead suppress sugar consumption. In each test, we offered starved *D. melanogaster* (*n* = 10 to 15 females/test) 50 mM glucose water solution dyed blue for 15 min in presence or absence of an odor: one group of flies was exposed to volatile AITC (1:500 vol/vol; flies could not contact the odor solution), and the control group was exposed to the mineral oil solvent (supplementary fig. S1c, Supplementary Material online; flies could not contact the odor source). We calculated a feeding score/test (each vial constitutes a biological replicate) based on the amount of blue dye in the abdomen of flies. Control flies fed less when volatile AITC was present than in the presence of the solvent (one-sample signed rank tests on normalized data, *P* < 0.001; Fig. 4b). *Or42a^−/−^* mutant flies fed less in the presence of AITC volatiles as well (*P* < 0.05), but their feeding scores were higher than those of the control group (*P* < 0.001, Mann–Whitney *U* test; Fig. 4b). Altogether, these results show that Or42a mediates aversion to AITC volatiles in two different behavioral contexts.

While we showed that Or42a-positive OSNs mediate behavioral aversion to AITC (Fig. 4), Dweck et al. (2016) found that these OSNs mediate attraction to some fruit/fermentation volatiles. We thus tested whether Or42a also mediates behavioral attraction to such compounds in our experimental setup/s using γ-hexalactone, which has been reported to activate Or42a-positive OSNs (Dweck et al. 2016). In the positional olfactory assay, genetic background control and wild-type Canton-S flies, but not *Or42a^−/−^* mutants, were attracted γ-hexalactone 1:10 vol/vol at various time points (one-sample signed rank tests, *P* < 0.05 for both lines; supplementary fig. S2d and supplementary file S4, Supplementary Material online). Similarly, in consumption assays, Canton-S flies increased their feeding in the presence of volatile γ-hexalactone 1:50 vol/vol, but this effect was lost in *Or42a^−/−^* mutants (supplementary fig. S2j, Supplementary Material online). Additionally, we found that volatile γ-hexalactone, contrary to what we observed in tests with AITC, does not immobilize flies (supplementary fig. S2k, Supplementary Material online). Thus, our behavioral assays show that volatile γ-hexalactone attracts *D. melanogaster* via *Or42a* and is harmless, in line with previous results (Dweck et al. 2016).

Given that Or42a OSNs mediates both repellence to AITC and attraction to γ-hexalactone (Fig. 4 and supplementary fig. S2a to e and j, Supplementary Material online), we hypothesized that additional OSNs contribute to mediate these contrasting behavioral responses. We performed exhaustive SSR from antennae and maxillary palps and found that pb1a OSNs were the only ones activated by γ-hexalactone among basiconic, intermediate, and trichoid sensilla (supplementary fig. S2l, Supplementary Material online). Due to technical limitations, we were unable to study antennal coeloconic sensilla, but Oh et al. (2021) reported that this odor activates Or35a OSNs in ac3b (Yao et al. 2005). In contrast, AITC activated several OSNs within the above-mentioned sensilla types, including Or7a OSNs (>75 spikes/s, Fig. 3b), which are housed in ab4a (Lin et al. 2015). Thus, we investigated whether *Or7a* and *Or35a* could contribute to mediate the observed behavioral repellence to AITC and attraction to γ-hexalactone, respectively. Compared to genetic background controls, *Or7a^−/−^* flies showed less aversion to AITC in the positional olfactory assay (supplementary fig. S2f and g, Supplementary Material online), while *Or35a^−/−^* flies lost the attraction to γ-hexalactone (supplementary fig. S2h and i, Supplementary Material online). Thus, not only Or42a but Or35a and Or7a are also necessary to mediate attraction/repellence behaviors to these odorants: activation of both Or42a and Or7a OSNs by AITC might mediate aversion, while activation of both Or42a and Or35a OSNs by γ-hexalactone might mediate attraction. Our findings thus support a combinatorial hypothesis of odor coding with respect to these ligands and indicate that the valence of Or42a-mediated behavioral responses is context-dependent (supplementary fig. S2m, Supplementary Material online).

### Pb1a-like OSNs in Brassicales Specialists Evolved Broadened Sensitivity to ITCs

We next investigated how the evolutionary transitions from microbe-feeding to herbivory have affected the odor tuning of OSNs, using the reference species *D. melanogaster* as well as species within *Scaptomyza* (the microbe-feeding *Scaptomyza hsui* and *S. pallida*) and the herbivorous Brassicales specialists *S. flava* and *S. montana* (Kim et al. 2021; Peláez et al. 2023). We investigated whether *Scaptomyza* species have pb1-like sensilla homologous to *D. melanogaster* pb1 and, if so, the extent to which they respond to a broader range of ITC compounds, since Brassicales plants release many different ITCs upon wounding (MacLeod et al. 1989; Cuellar-Nuñez et al. 2022).

**Fig. 5.**
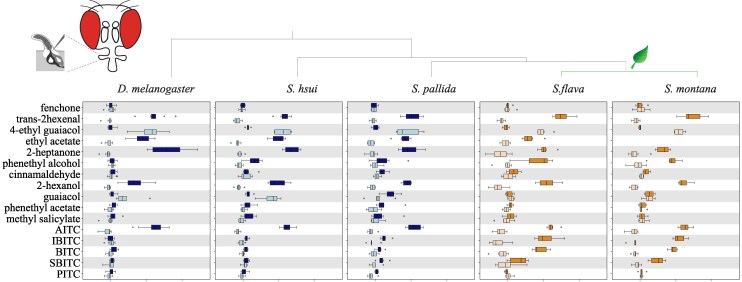
Maxillary palp pb1-like sensilla of the mustard plant specialist *Scaptomyza* species have an expanded ITC sensitivity range. SSRs from maxillary palp pb1 OSNs of *D. melanogaster, S. hsui, S. pallida, S. flava,* and *S. montana*. Stimuli (1:100 vol/vol) included diagnostic chemicals used to identify Ors in *D. melanogaster* (see the Materials and Methods section), fruit volatiles, green leaf volatiles, and Brassicales plant-derived ITCs (*n* = 6 to 9 from three to four animals/species). pb1 and pb1-like sensilla housed two OSNs, labeled “a” (darker color) and “b” (lighter color). See the Materials and Methods section for sensilla classification and the supplementary figs. S3 and S4, Supplementary Material online, for functional characterization of pb2 and pb3. While pb1a OSNs from all species responded to AITC, pb1a-like OSNs from the mustard plant specialists *S. flava* and *S. montana* additionally responded to other ITC compounds. Mustard specialization occurred at the clade leading to the common ancestor of these two species, denoted by the leaf cartoon. Because sensilla with extremely small spike amplitudes were excluded from analysis, additional unidentified palp basiconic sensilla may exist in *Scaptomyza*. PITC, phenethyl ITC. The structure of ITCs is shown in supplementary fig. S5, Supplementary Material online.

We first validated our methods for functional characterization of sensilla. We confirmed the presence of three different types of palp sensilla (pb1, pb2, and pb3) in this species (Fig. 5 and supplementary figs. S3 and S4, Supplementary Material online) using compounds that serve as diagnostic for the three sensilla types found in the maxillary palps of *D. melanogaster* (see the Materials and Methods section for details), various other plant-derived volatiles, and several Brassicales-derived ITCs. As we observed before, *D. melanogaster* pb1a OSNs responded to AITC (>120 spikes/s), but not to the other ITCs tested (<10 spikes/s, Fig. 5). We next characterized OSNs in pb sensilla in the four *Scaptomyza* species and used the spike rate to determine if data clustered by sensilla type (Fig. 5, supplementary figs. S3 and S4, Supplementary Material online). In all *Scaptomyza* species, sensilla fell into three functional classes, two of which were functionally similar to *D. melanogaster* pb1 and pb2 (we termed them pb1-like and pb2-like). The third class was functionally different to any *D. melanogaster* palp sensilla type and clustered separately (termed pb3-like, supplementary figs. S3 and S4, Supplementary Material online). As in *D. melanogaster*, AITC activated pb1a-like sensilla in all four *Scaptomyza* species (Fig. 5), but several other ITCs, including isobutyl ITC (IBITC), BITC, and *sec*-BITC (SBITC), additionally activated *S. flava* and *S. montana* pb1a-like OSNs (Fig. 5).

We also conducted dose-responses to various odorants from pb1a/pb1a-like sensilla of the microbe-feeding species (*D. melanogaster*, *S. hsui*, and *S. pallida*) and the Brassicales specialists (*S. flava* and *S. montana*). On the whole, responses increased with increasing odorant concentration (supplementary fig. S5a, Supplementary Material online). For comparing odor sensitivity across species, we calculated the odorant concentration required to elicit a biological response halfway between the baseline and the maximum (50% effective concentration, EC_50_; supplementary fig. S5d, Supplementary Material online). In agreement with its herbivore habit, the EC_50_ for the fruit odor γ-hexalactone was higher in *S. flava* than in the microbe-feeding *S. hsui* (*S. flava* had even lower sensitivity to this odorant than the herbivore *S. montana*; supplementary fig. S5b and d, Supplementary Material online). The EC_50_ for trans-hexenal was lower in the two herbivores species than in *D. melanogaster* or *S. hsui* (supplementary fig. S5d, Supplementary Material online), and *S. flava* was even more sensitive to this odorant than *S. montana* (supplementary fig. S5c, Supplementary Material online). Similarly, the EC_50_ for AITC was lower in all the *Scaptomyza* species, but the two mustard specialists had similar EC_50_ for all the ITCs tested (supplementary fig. S5d, Supplementary Material online, right). Overall, all these findings suggest that microbe-feeding species exhibit higher sensitivity to the fruit odor γ-hexalactone, whereas Brassicales specialists show heightened sensitivity to electrophilic trans-2-hexenal and ITCs.

### *Or42a* Is Triplicated in the Genome of *S. flava* and Is Highly Expressed in the Maxillary Palps

**Fig. 6.**
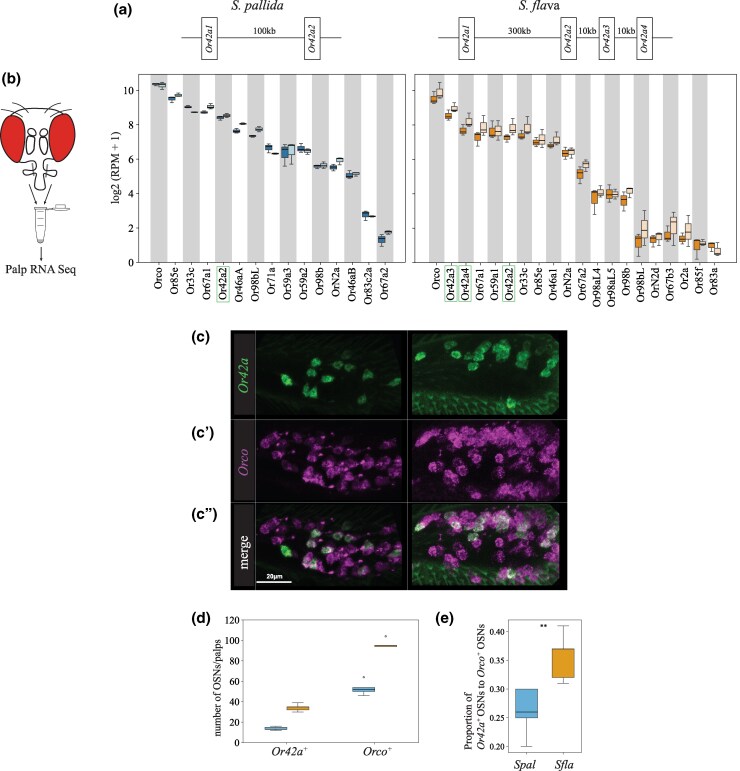
High expression of *Or42a* paralogs and over-representation of *Or42a*-positive OSNs in the maxillary palp of *S. flava*. a) Schematic of *Or42a* syntenic regions in the genomes of the microbe-feeding *S. pallida* and the mustard plant specialist *S. flava*, with a gene triplication in the *S. flava* genome at the syntenic region of *S. pallida Or42a2* (*S. flava Or42a2*, *S. flava Or42a3*, and *S. flava Or42a4*). b) Maxillary palp RNA-seq of *S. pallida* and *S. flava Or*s (*n* = 3 replicates/sex and species). *Or*s with median values of the log2 (RPM +1) < 1 (*n* = 3) were excluded. *S. pallida* expresses only one copy of Or42a, while the specialist *S. flava* expresses three copies. c to c") Representative images of hybridization chain reaction RNA FISH from the maxillary palps of *S. pallida* and *S. flava* showing *Or42a*-positive OSNs (c, green), *Orco*-positive OSNs (c', magenta), and the merged signals (c", white indicates co-localization of *Or42a*-positive OSNs and *Orco*-positive OSNs). Or42a is expressed in OSNs. Scale bar: 20 µm. d and e) Number of *Or42a*-positive OSNs and *Orco*-positive OSNs in the maxillary palps of *S. pallida* (blue boxes) and *S. flava* (orange boxes) (d) and the ratio between them (e). Mann–Whitney *U* tests, **P < 0.01; *n* = 5 animals/species. *S. flava* has more Or42a-positive OSNs and more Orco-positive OSNs than *S. pallida*.

Genomic analysis across the *Scaptomyza* genus revealed a duplication of *Or42a* in the lineage leading to all known *Scaptomyza*, while the *D. melanogaster* outgroup had a single *Or42a* homolog (supplementary fig. S6a, Supplementary Material online). Notably, syntenic analysis indicated that *S. flava* has a species-specific tandem triplication in one of the *Or42a* duplicates, resulting in three tandem paralogs, which we named *Or42a2*, *Or42a3*, and *Or42a4* (Fig. 6a and supplementary fig. S6a and b, Supplementary Material online). In contrast, *S. montana*, *Scaptomyza graminum, S. pallida*, and *S. hsui* retained only two *Or42a* paralogs.

Given these differences in the Or42a gene copy number across species, we conducted species- and sex-specific RNA transcriptome analyses of OSNs in the maxillary palps of the microbe-feeding *S. pallida* and the mustard plant specialist *S. flava*. We confirmed the expression of *S. pallida Or42a* and *S. flava Or42a2-4* in these organs (Fig. 6b and supplementary fig. S7, Supplementary Material online). Interestingly, the *S. flava Or42a* paralogs were each expressed at levels comparable to those of other *Or* genes, such as *Or33c*, the homolog of which is expressed in pb2a OSNs in *D. melanogaster* (supplementary fig. S7, Supplementary Material online). We found that *S. pallida Or42a2* and *S. flava Or42a2-4* were expressed in the respective maxillary palp of each species, whereas *S. pallida Or42a1* and *S. flava Or42a1* were not expressed (Fig. 6b).

### The Brassicales-Specialist *S. flava* Has More *Or42a*-Positive Olfactory Sensory Neurons

Given that pb1a-like OSNs likely express *Or42a* (Fig. 6b), we hypothesized that the number of *Or42a*-positive OSNs is higher in the specialist species. To test this, we quantified the number of *Or42a*-positive OSNs in *S. flava* and *S. pallida* using hybridization chain reaction RNA fluorescent in situ hybridization (FISH). We designed a *S. flava Or42a* RNA probe based on the conserved sequence region of *S. flava Or42a2*, *3*, and *4*, as the high sequence similarity among these paralogs prevented the design of paralog-specific probes. We found that the maxillary palps of *S. flava* contained more *Or42a*-positive OSNs and more *Orco*-positive OSNs (Orco is a highly conserved co-receptor necessary for odorant receptor olfactory function, Larsson et al. 2004) than those of *S. pallida* (Fig. 6c and d). Because all maxillary palp OSNs express Orco (Larsson et al. 2004), the number of Orco-positive OSNs represents the total number of palp OSNs. Importantly, the ratio of the total number of *Or42a*-positive OSNs to the total number of *Orco*-positive OSNs was higher in *S. flava* (Fig. 6e). These results indicate that *S. flava* has not only more *Or42a*-positive OSNs in the maxillary palps but also a greater proportion of *Or42a*-positive OSNs relative to the overall number of OSNs. Accordingly, we predicted that the number of pb1-like sensilla would be also overrepresented in mustard specialists. To test this, we generated functional anatomical maps of sensilla on the anterior part of the maxillary palps of the *Scaptomyza* species (and of *D. melanogaster* for comparison) using diagnostic chemicals (supplementary figs. S8 and S9, supplementary file S10, Supplementary Material online). While *D. melanogaster* had a relatively randomized distribution of sensilla on the palps, consistent with previous reports (de Bruyne et al. 1999), all four *Scaptomyza* species exhibited a more organized sensilla pattern, with pb1-like, pb2-like, and pb3-like, respectively, located medially, distally, and proximally, as reported in *Drosophila mojavensis*, which is more closely related to all *Scaptomyza* spp. than *D. melanogaster* (Crowley-Gall et al. 2016). We then quantified the number of each sensilla type across species and found that both *S. flava* and *S. montana* have a larger number of pb1-like than pb2-like or pb3-like sensilla, while *D. melanogaster* and the other two microbe-feeding *Scaptomyza* species had similar proportions of each sensilla type (supplementary figs. S8 and S9, Supplementary Material online). These findings, showing a triplication of Or42a, along with an expansion in the number of Or42a-positive OSNs and pb1a-like sensilla, are in line with the enhanced capacity of Brassicales-specialist *Scaptomyza* species to detect volatile ITCs.

### Paralog-Specific Functional Evolution of the Olfactory Receptor Or42a

**Fig. 7.**
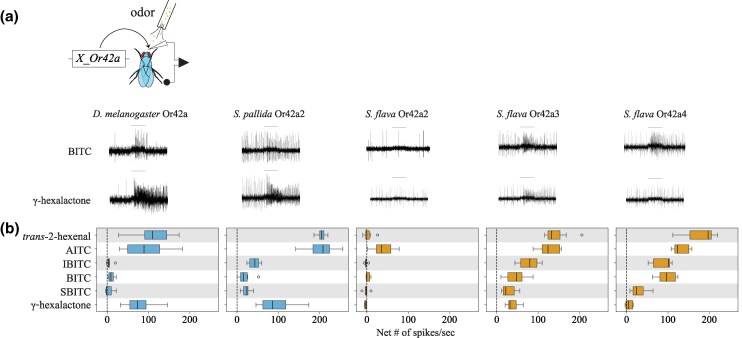
Functional characterization of the olfactory receptor Or42a from *D. melanogaster*, *S. pallida*, and *S. flava.* a) Representative SSR traces from *D. melanogaster* at1 sensilla OSNs expressing species-specific *Or42a* under the control of *Or67d ^Gal4^* (fly genotype: *UAS-Or42a; Or67d ^Gal4^*) in response to stimulation with BITC and the fruit odor γ-hexalactone. The horizontal bars above records indicate the onset and duration (1 s) of the stimulation. b) Responses of at1 OSNs (*n* = 6 to 8 sensilla from three to four animals/genotype) expressing species-specific *Or42a* upon stimulation with trans-2-hexenal (a general leaf odor released upon leaf mechanical damage such as crushing), various ITCs produced by mustard plants (AITC, IBITC, BITC, and SBITC), and γ-hexalactone. Orthologs from all species respond to AITC, while *S. flava* Or42a3 and *S. flava* Or42a4 additionally respond to various ITCs. In contrast, only paralogs from microbe-feeding species show strong responses to γ-hexalactone.

We next investigated whether the increased sensitivity of *S. flava* pb1a-like OSNs to ITCs (Fig. 5) also resulted from changes in the odor tuning of the Or42a triplicates. To test this, we expressed *D. melanogaster* Or42a*, S. pallida* Or42a2, and *S. flava* Or42a2-4 in *D. melanogaster* antennal trichoid 1 (at1) OSNs (in the background of a null mutation for *Or67d*, the at1 cognate receptor; Kurtovic et al. 2007) and conducted functional analysis. We found that γ-hexalactone, trans-2-hexenal, and AITC strongly activated OSNs expressing *D. melanogaster* Or42a (>79 spikes/s) or *S. pallida* Or42a2 (>96 spikes/s), while the other ITC compounds evoked much weaker responses from these two orthologs (<11 and 18 spikes/s, respectively; Fig. 7a and b). OSNs expressing *S. flava* Or42a3 or *S. flava* Or42a4 were also very sensitive to AITC and trans-2-hexenal but additionally responded to IBITC and BITC (>87 and 46 spikes/s, respectively; Fig. 7b), consistent with the odor response profiles of *S. flava* pb1a OSNs (Fig. 5). Notably, OSNs expressing *S. flava* Or42a4 showed only small responses to γ-hexalactone (<10 spikes/s), and *S. flava* Or42a2 was only activated by AITC (Fig. 7b). These results are in line with the broader ITC sensitivity of *S. flava* pb1a OSNs compared to that of *D. melanogaster* pb1a and *S. pallida* pb1a-like OSNs. Altogether, our findings reveal paralog-specific functional evolution of Or42a triplicates in *S. flava*, wherein different paralogs evolved distinct sensitivities to different ITCs and fruit odors.

### AlphaFold2-Led Screening with Ectopic Expression of *S. flava* Or42a Reveals the Molecular Changes Underlying Changes in Odor Sensitivity

We next investigated which amino acid substitutions in *S. flava* O42a4 may have led to the gain of sensitivity to BITC and the decreased sensitivity to γ-hexalactone (there are 32 amino acid differences between *S. flava* Or42a3 and *S. flava* Or42a4, supplementary fig. S10a, Supplementary Material online). To explore the structural basis of these functional differences, we predicted the 3D structures of *S. flava* Or42a3 and *S. flava* Or42a4 and aligned the resulting models in 3D space using PyMol (Rambaut 2009; Katoh and Standley 2013; Stamatakis 2014; Jumper et al. 2021; Mirdita et al. 2022; Benton and Himmel 2023; Himmel et al. 2023).

**Fig. 8.**
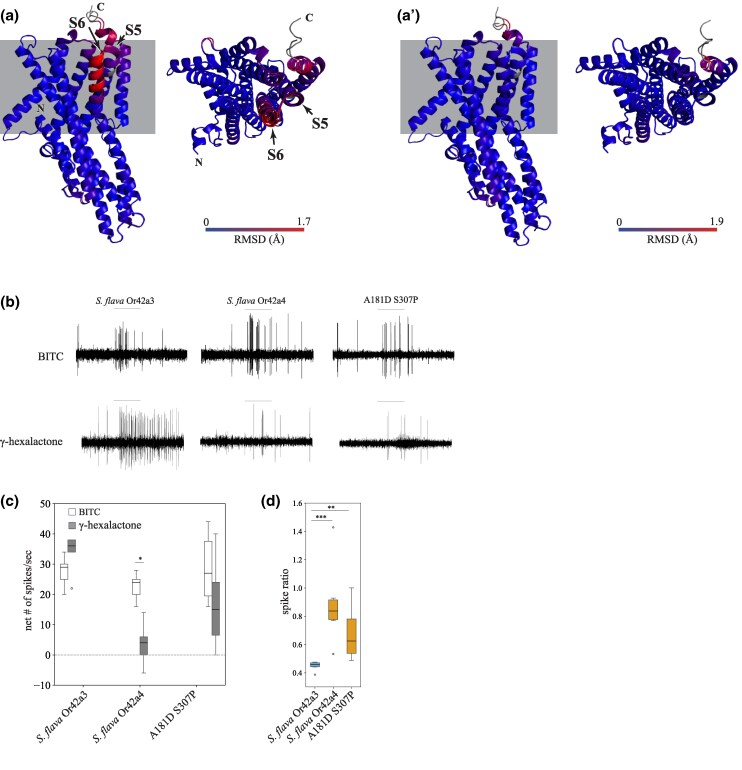
Two amino acids are critical for changing the sensitivity of *S. flava* paralogs from fruit volatiles to ITCs. a and a') The 3D alignment of *S. flava* Or42a3 and *S. flava* Or42a4 predicted by AlphaFold2 (a), and 3D alignment of *S. flava* Or42a4 and a chimeric Or42a with two amino acid substitutions (A181D and S301P) in the background of *S. flava* Or42a3 (a'). The root mean square deviation is visualized with a color gradient from blue (low) to red (high) in both the side (left) and the top view (right). The upper and lower sections of the side view represent the extracellular and the intracellular regions separated by the cell membrane (gray rectangles), respectively. b to d) Representative SSR from *D. melanogaster* at1 sensilla expressing heterologous *S. flava* Or42a3, Or42a4, and the chimera (genotype: *UAS-Or42a/CyO; Or67d^Gal4^*) upon stimulation with BITC and γ-hexalactone (b), at1 population responses to BITC (white bars) and to γ-hexalactone (gray bars) (c, *n* = 6 to 10 sensilla from three to four animals/genotype; *P < 0.05, Mann–Whitney *U* tests), and spike ratio [d (response to BITC)/(response to BITC + response to γ-hexalactone); **P < 0.01, ***P < 0.001, Kruskal–Wallis ANOVA followed by Dunn's multiple comparisons]. The odor tuning of the chimera, with just two amino acid substitutions, recapitulates that of *S. flava* Or42a3.

We first confirmed that the predicted local distance difference test scores for the *S. flava* Or42a3 and *S. flava* Or42a4 structures were sufficiently high to ensure confidence in the 3D predictions, except for the N-terminal and C-terminal regions (supplementary fig. S10b, Supplementary Material online). The most striking 3D structure difference between *S. flava* Or42a3 and *S. flava* Or42a4 was in the S5 and S6 helices in the transmembrane region (∼1.7 Å root mean square deviation, Fig. 8a and a' and supplementary fig. S11a to c, Supplementary Material online), which is reported to contain the ligand binding pockets of Ors (Del Mármol et al. 2021; Wang et al. 2024; Zhao et al. 2024). We substituted each of the 32 amino acids in *S. flava Or42a4* individually with the corresponding residues from *S. flava* Or42a3 in silico, predicted the 3D structures of the chimeras, and aligned them with *S. flava* Or42a4 in 3D space until the local structural differences were resolved. Remarkably, the substitutions of A181D and S307P in *S. flava* Or42a3 (hereafter referred to as A181D S307P) reduced the root mean square deviation to approximately 0.1 Å in the S5 and S6 helices when aligned with *S. flava* Or42a4, indicating that these two mutations are likely to explain the local structural differences (Fig. 8a and a' and supplementary fig. S11a and c, Supplementary Material online).

We then used the *D. melanogaster* at1 empty neuron system to investigate whether these two amino acid substitutions could account for the differences in odor sensitivity between *S. flava* Or42a4 and *S. flava* Or42a3. The A181D S307P variant and the two *S. flava* paralogs showed similar moderate responses to BITC (Fig. 8b and c). However, the BITC to γ-hexalactone response ratios of *S. flava* Or42a4 and A181D S307P were not different from each other but were both higher than the response ratio of *S. flava* Or42a3 (Fig. 8d). These findings suggest that the two amino acid substitutions in *S. flava* Or42a4 are sufficient to increase the sensitivity to BITC relative to γ-hexalactone. This effect was observed in flies carrying the heterozygous genotype A181D S307P/+ but not in flies with the homozygous A181D S307P genotype (Compare Fig. 8c and d with supplementary fig. S12, Supplementary Material online), possibly due to response saturation or dominant-negative effects. In summary, our findings demonstrate that the A181D and S307P substitutions in *S. flava* Or42a4 are critical for shifting the receptor's sensitivity from fruit odorants to ITCs.

## Discussion

In this study we addressed three major questions: (1) Do nonspecialist insects use olfaction to detect and avoid toxic ITC compounds released by mustard plants? (2) Was this ancestral olfactory capability co-opted and expanded in mustard plant specialists for facilitating host plant detection? (3) What molecular changes underlie the olfactory receptor sensitivity shift from fruit odors to host plant-derived toxins? Answering these questions is important for understanding which molecular changes in the chemoreceptors of ancestral nonspecialists enabled adaptation to toxic ecological niches, a central issue in the evolution of herbivory. We found that plant-derived ITCs are detrimental to microbe-feeding *D. melanogaster* through volatile exposure and that the olfactory receptor Or42a is necessary for its detection and for behavioral aversion. To our knowledge, Or42a is the first ITC olfactory detector reported for *D. melanogaster*. Additionally, in the mustard plant specialist *S. flava*, homologous olfactory receptors are triplicated and have an expanded ITC sensitivity range, accompanied by an increase in the number of ITC-detecting OSNs. Finally, we discovered that two amino acid changes are sufficient to shift the odor sensitivity of these paralogs from fruit odors to ITC volatiles.

### The Plant-Derived Volatile AITC Is Toxic to *D. melanogaster* and Its Detection and Avoidance Is Mediated by *Or42a*-positive OSNs

Plants have evolved a diverse array of specialized metabolites, including electrophilic ITCs, that repel or intoxicate insects (Ibanez et al. 2012; Noge and Becerra 2015; Tocmo et al. 2021). ITCs are not only detected by contact (Bell et al. 2018), but many mustard plant specialists, such as the diamondback moth *Plutella xylostella* (Liu et al. 2020) and *S. flava* (Matsunaga et al. 2022), also detect these compounds via olfaction. However, it was unclear whether nonspecialists such as *D. melanogaster* have evolved strategies to detect these toxic compounds using olfaction. We found that the olfactory receptor Or42a is necessary for OSN responses in the sensilla where it is expressed (palp basiconic 1a sensilla, Fig. 3c). Furthermore, this Or was necessary for inducing olfactory aversion to AITC in two different behavioral contexts (Fig. 4 and supplementary fig. S2a, Supplementary Material online). Thus, in *D. melanogaster*, Or42a works in combination with the “wasabi taste receptor” TrpA1 and Painless (Al-Anzi et al. 2006; Kang et al. 2010; Mandel et al. 2018), and possibly with other Ors (see next paragraph), to facilitate adaptive behavioral avoidance of these chemicals. It remains to be tested whether other nonspecialist organisms across phyla also possess olfactory sensors tuned to volatile ITCs.

We found that Or42a is also necessary for behavioral attraction to the fruit-derived volatile γ-hexalactone (supplementary fig. S2d, e, and j, Supplementary Material online). How does a single olfactory channel mediate aversion to AITC while also driving attraction to γ-hexalactone? Our experiments suggest that simultaneous activation of Or42a and the “generalist” OR7a (which is housed in OSNs in ab4 and respond to aversive odorants; Lin et al. 2015) could mediate aversion to AITC (Fig. 4 and supplementary fig. S2f, g, and m, Supplementary Material online). Similarly, simultaneous activation of Or42a and Or35a (which is housed in ac3b, responds to γ-hexalactone and mediates attraction to yeast odors; Yao et al. 2005; Oh et al. 2021) could mediate attraction to γ-hexalactone (supplementary fig. S2h, i, and m, Supplementary Material online). Future investigations at the circuit level, particularly on the role of interglomerular interactions via local neurons in the insect primary olfactory center (Haverkamp et al. 2018), should further elucidate the neural mechanisms mediating these behavioral responses of opposite valence.

### Duplication and Functional Evolution of the Olfactory Receptors *Or42a* and *Or67b* in *Scaptomyza* Mustard Plant Specialists

Brassicales specialist need to detect a wide range of ITC compounds for effective host plant location, as these plants release species-specific volatile ITCs at particular ratios and concentrations (Wu et al. 2021). We previously reported that *S. flava* also has triplicated and positively selected *Or67b* copies (Matsunaga et al. 2022). The Ors encoded by these paralogous *Or67b* copies respond to aromatic and some aliphatic ITCs in a paralog-specific manner, while the *D. melanogaster* and *S. pallida* Or67b single copies did not respond to any volatile ITC compound (Matsunaga et al. 2022). However, all three *S. flava* Or67b paralogs showed poor responses to organosulfur ITCs, including AITC. In this study, we found that *S. flava* also expresses tandem triplicates of the *Or42a*s (*Or42a2*, *Or42a3*, and *Or42a4*; Fig. 6a and supplementary fig. S6, Supplementary Material online; Nishimura et al. 2024). Furthermore, OSNs housed in the pb1a-like sensilla of the mustard specialist species responded to many volatile ITCs, including AITC (Fig. 5 and supplementary fig. S5, Supplementary Material online). Thus, the gene duplications and amino acid substitutions of Or42a, along with those of Or67b, both likely play an important role in enabling Brassicales plant specialist *Scaptomyza* species to detect a wide range of ITCs. Furthermore, mustard plant specialization is likely aided by the losses of genes encoding four olfactory receptors that detect fermentation odors in *D. melanogaster* and are necessary for attraction to these odors, and by the loss of an ancestral olfactory receptor (*Or7a*, housed in ab4a) that mediates aversion to AITC (supplementary fig. S2f and g, Supplementary Material online; Goldman-Huertas et al. 2015).

What is the functional relevance of ITC-sensitive Ors expressed in two different olfactory organs? Or67b in *S. flava* is primarily expressed in the antennae (Matsunaga et al. 2022), whereas Or42a in *S. flava* and the other drosophilids is expressed in the maxillary palps (Fig. 6b). In *D. melanogaster*, maxillary palps OSNs have lower sensitivity thresholds to certain host-related compounds compared to antennal OSNs (Dweck et al. 2016). Indeed, *S. flava* Or42a paralogs are much more sensitive to AITC than the Or67b paralogs (compare Fig. 5 with Fig. 4 in Matsunaga et al. 2022). Odor response redundancy between antennal and maxillary palp Ors could have evolved to further underpin olfactory orientation over both long and short distances in drosophilids (Dweck et al. 2016). Given the proximity of Or42a OSNs to the mouthparts, their activation could potentially modulate feeding behaviors, as suggested in *D. melanogaster* (Shiraiwa 2008). *S. flava* and *S. montana* females feed on the juice that seeps into the leaf wounds they create in Brassicales plants before oviposition (Peláez et al. 2022). Given this stereotyped feeding behavior, while the activation of contact chemoreceptors by ITCs could help females assess the suitability of an oviposition site through taste, flies may also be aided by maxillary palp olfactory activation even before tasting the plants.

Although the OSNs in pb1a sensilla of the two mustard specialist species have a broad ITC response range (Fig. 5), *S. flava* has triplicated *Or42a*s but *S. montana* has only one copy (supplementary fig. S6, Supplementary Material online). This suggests that both the mustard plant specialization and the mutations underlying the expanded ITC sensitivity range of Or42a preceded the triplication of *Or42a*. What is then the adaptive value of the *Or42a* triplication? We observed that *S. flava* pb1a OSNs, which express at least one of the three triplicated Or42as (Fig. 6a and b), exhibited reduced sensitivity to fruit-borne γ-hexalactone in comparison with *S. montana* pb1a OSNs (supplementary fig. S5, Supplementary Material online). Only *S. flava* Or42a4 acquired the two key mutations that increased the ITC to γ-hexalactone response ratio, as evidenced by the weaker γ-hexalactone response of the chimeric Or compared to that of *S. flava* Or42a3 (Fig. 8). In agreement with these observations, structural alignment of the 3D models predicted by AlphaFold2 revealed that *S. montana* Or42a2 aligned well with *S. flava* Or42a3, but not with *S. flava* Or42a4 (supplementary fig. S11d, Supplementary Material online). Based on these findings, we hypothesized the following sequence in the evolution of Or42a (supplementary fig. S13, Supplementary Material online): (1) mustard specialization and broadening of ITC sensitivity in the ancestral drosophilid Or42a that was already sensitive to some ITCs like AITC, (2) speciation and triplication of Or42a in *S. flava* but not in *S. montana*, and (3) relaxation of evolutionary constraints due to gene duplications, allowing *S. flava* Or42a4 to reduce its sensitivity to γ-hexalactone. It was suggested that *S. flava* has a broader host range (Maca 1972; Martin 2004) than *S. montana*, which requires indolic glucosinolates to use plants as hosts (Gloss et al. 2017). Thus, although mustard specialization preceded the Or42a duplication, we hypothesize that gene duplication was an important event in driving adaptation to new ecological niches in herbivorous *Scaptomyza*.

### Evolution of Specialized Olfactory Receptors Is Coupled With Expansion of Maxillary Palp Sensilla and Its Associated Olfactory Sensory Neurons

We found that OSNs housed in pb1a sensilla from the mustard plant specialists *S. montana* and *S. flava* respond to a broad range of ITC compounds (Fig. 5 and supplementary fig. S5, Supplementary Material online) and that this sensilla type was numerically expanded in these two species (supplementary figs. S6 and S7, Supplementary Material online). Concomitantly with this, we discovered an increase in the number of *Or42a*-positive OSNs in *S. flava* compared to *S. pallida* (Fig. 6). Similar increases in OSNs that detect odors that bear species-specific biological significance have been reported in several *Drosophila* species. For instance, the noni fruit specialist *Drosophila sechellia* and the seasonal specialist of “screw pine” (*Pandanus* spp.) fruits *Drosophila erecta* both show an increase in the number of *Or22a-*positive OSNs (Dekker et al. 2006; Linz et al. 2013; Auer et al. 2020). In *D. sechellia*, these OSNs enhance odor tracking by reducing adaptation in second-order projection neurons (Takagi et al. 2024). We hypothesize that the increase in the number of *Or42a*-positive OSNs in *S. flava* may similarly contribute to enhance odor sensitivity and tracking during host plant finding, although this remains to be investigated.

### Insight into the Binding Pocket of *S. flava* Olfactory Receptor Or42a Paralogs

Our results demonstrate that the Or42a paralogs from microbe-feeding species show strong responses to both AITC and γ-hexalactone (Fig. 7), while the paralogs from the herbivorous *S. flava* show a notable shift in olfactory sensitivity, which aligns with the fact that this species has undergone a full transition to herbivory. For example, *S. flava* Or42a3 showed a moderate response to γ-hexalactone (although about an order of magnitude lower than that of the Or42a from the microbe-feeding *S. pallida*), and *S. flava* Or42a4 was even less sensitive (Fig. 7). Furthermore, *S. flava* Or42a4 had a relatively higher sensitivity to BITC compared to Or42a3 (Fig. 7). To explore the mechanisms underlying this shift from fruit detector to ITC detector, we employed computational and functional approaches to identify the amino acid substitutions responsible for the differential odor sensitivity of *S. flava* Or42a3 and Or42a4.

Our AlphaFold2-led screening, combined with site-directed mutagenesis and electrophysiological studies, identified two key substitutions in the transmembrane region of Or42a4, A181D and S307P, which are critical for the odor sensitivity switch (Fig. 8). Proline is a secondary structure breaker (Chou and Fasman 1978; Levitt 1978), and the substitution of serine by proline (S307P) likely influenced the conformation change of the binding pocket, altering the protein structure, polarity, and hydrophobicity. Similarly, the substitution of alanine by aspartic acid (A181D) could alter protein polarity and hydrophobicity. Thus, these two amino acid changes likely account for the notable change in ligand sensitivity. However, this change in ligand sensitivity was observed only in flies heterozygous for A181D and S307P (Or67d^Gal4^; UAS-A181D S307P/+), but not in homozygous flies (compare Fig. 8c and d with supplementary fig. S12, Supplementary Material online). Given that homozygous flies express A181D and S307P more strongly than the heterozygous ones, it is possible that the OSNs' response to both odorants reached saturation in the former, obscuring differences in spike ratios. Alternatively, excessive expression of A181D and S307P may have altered the 3D structure of the heteromeric protein, possibly through dominant-negative effects between the copies, reverting it closer to the original *S. flava* Or42a3 conformation and restoring the original binding pocket. Nonetheless, the partial rescue we observed in heterozygous flies indicates that A181D and S307P are critical for altering the binding pocket structure, enabling the Or to better accommodate BITC instead of γ-hexalactone. Although the remaining amino acid substitutions in Or42a4 might alter e.g. signal transduction and/or receptor stability, it is remarkable that only two substitutions out of 32 amino acid differences between paralogs were sufficient to shift ligand sensitivity.

## Conclusions

Taken together, our findings reveal that nonherbivorous, microbe-feeding insects like *D. melanogaster* have evolved promiscuous olfactory sensory mechanisms that allow them to detect and avoid specialized plant-derived volatile electrophilic toxins, such as ITCs. In contrast, Brassicales plant specialists like *S. flava* not only have physiological adaptations to detoxify these toxic compounds but also have undergone significant evolutionary sensory adaptations for aiding host plant location, including turning shifts and expansions of specialized ITC olfactory receptors. Furthermore, our use of AlphaFold2, followed by site-directed mutagenesis and electrophysiology, identified critical amino acid changes for the evolution of these specialized odorant receptors that we confirmed experimentally. Thus, ancestral Ors that mediate toxin aversion in generalist species can be co-opted and diversified in derived specialists through gene duplications and tuning shifts facilitated by relatively few amino acids substitutions.

## Materials and Methods

### Fly Husbandry

*Drosophila melanogaster* was reared on cornmeal medium. Microbe-feeding *S. pallida* (*S. pallida* subgenus *Parascaptomyza*) and *S. hsui* (*S. hsui* subgenus *Hemiscaptomyza*) were reared in cornmeal molasses media covered with a mixture of Carolina biological supply instant *Drosophila* media (Burlington, North Carolina, USA) mixed with blended spinach leaves, and then covered with a layer of defrosted frozen spinach leaves. The obligate leaf-miners *S. flava* and *S. montana* (subgenus *Scaptomyza*) were cultivated on potted fresh laboratory-grown *A. thaliana* Col-0. Isofemale lines of microbe-feeding *S. hsui* and *S. pallida*, as well as the herbivorous *S. montana,* were collected along Strawberry Creek on the UC Berkeley Campus in Berkeley, California, USA (Kim et al. 2021), and a line of *S. flava* was collected from a meadow near Dover, New Hampshire, USA. All species were kept at 23 ± 2 °C and 60% relative humidity in a 10:14 h light–dark cycle under fluorescent lights. The following lines (stock #) were obtained from the Bloomington Drosophila Stock Center: *Or42a^−/−^* (60821), *Or42a-Gal4* (9970), *w^1118^* (3605), *TrpA1^1^* (26504), *Or7a^/−^* (91811), and a genetic background control for the three *Or* null mutant lines (68384). The *Or35a^−/−^* line (10564) was obtained from the Korea Stock Center. The *Or67d^Gal4^* line was a gift from the laboratory of Barry J. Dickson.

### Single Sensillum Recordings

Fed female flies 1 to 5 days old were prepared for SSR as described by Matsunaga et al. (2022). Briefly, a silicon tube delivering a constant flow of charcoal-filtered air (16 ml/min, measured using a flowmeter, Gilmont Instruments, USA) was placed near the fly's head capsule, and the tip of the stimulation pipette (50 ml) was inserted into the constant air stream. The stimulation pipette contained a 0.5 cm × 5 cm piece of filter paper loaded with 20 µl of an odorant solution or the solvent control. A pulse of clean air (duration = 1 s) was delivered to the stimulus pipette using a membrane pump operated by a Stimulus Controller CS 55 (Syntech, Buchenbach, Germany). Sensilla identification was conducted using the following diagnostic odorants (as described in Dweck et al. 2016; Gonzalez et al. 2016), all >95% pure (Sigma-Aldrich, St. Louis, Missouri, USA): ethyl acetate (CAS# 141-78-6) for identifying *D. melanogaster* pb1a; AITC (CAS# 57-06-7) for identifying *Scaptomyza* pb1a-like OSNs; 4-ethylguaiacol (CAS# 2785-89-9) for identifying *D. melanogaster* and *Scaptomyza* pb1b and pb1-like OSNs; fenchone (CAS# 1195-79-5) for identifying *D. melanogaster* and *Scaptomyza* pb2a and pb2a-like OSNs; guaiacol (CAS# 90-05-1) for identifying *D. melanogaster* and *Scaptomyza* pb2b and pb2b-like OSNs; phenethyl acetate (CAS# 103-45-7) for identifying *D. melanogaster* pb3b, *S. flava* pb3b-like, and *S. montana* pb3b-like OSNs; and 2-heptanone for identifying *S. hsui* and *S. pallida* pb3a-like OSNs. All odorants were diluted in mineral oil (CAS# 8042-47-5) except γ-hexalactone (CAS# 695-06-7), which was diluted in dimethyl sulfoxide (CAS# 67-68-5) because it did not dissolve completely in mineral oil and sometimes produced response artifacts. Odorants were diluted to 1:100 vol/vol for stimulation unless otherwise noted. Supplementary file 1, Supplementary Material online, lists all the chemicals used in this study.

The “net number of spikes/second” was obtained by counting the number of spikes originating from the OSN of interest within a 0.5-s timeframe which started 0.2 s after the onset of stimulation. This count was then adjusted by subtracting the background spiking activity (# of spikes within a 0.5-s interval preceding the onset of the stimulation) and then doubled to represent the number of spikes/second. In all figures, unless otherwise stated, we represent the “control-subtracted net # of spikes/s” to odorant stimulation, calculated by subtracting the average net # of spikes/s in response to the solvent control (mineral oil or dimethyl sulfoxide) from the net # of spikes/sec evoked by each odorant stimulation. Control-subtracted spike data are compiled in supplementary file 2, Supplementary Material online. The BITC to γ-hexalactone spike ratio (Fig. 8d and supplementary fig. S12, Supplementary Material online) was calculated as: net # of spikes/s evoked by BITC/(net # of spikes/s evoked by BITC + net # of spikes/s evoked by γ-hexalactone). We used this denominator for the ratio because the control-subtracted net # of spikes upon γ-hexalactone stimulation occasionally produced negative values (likely a response to the solvent control).

Half maximal EC_50_ were calculated using *Quest Graph EC50 Calculator* (AAT Bioquest, Inc., 2025 March 4, https://www.aatbio.com/tools/ec50-calculator). We primarily used the two-parameter feature with normalization, where responses were normalized to the largest response within the same chemical-species pair, with the minimum set to 0. This approach was chosen because the lowest tested concentration (10^−5^) still elicited non-zero spike activity (>10) in some chemical-species pairs. The four-parameter method without normalization, in which the maximum and minimum responses were free parameters, was used in cases where the two-parameter method failed to fit a logistic regression (summarized in supplementary file 2, Supplementary Material online) or when analyzing SBITC data. For this odorant, the four-parameter method was necessary because the highest tested concentration (10^−2^) did not reach saturation (<100 spikes). When both the two-parameter and four-parameter methods failed due to lack of convergence at the lowest concentration (10^−5^), resulting in a calculated value of 0, we instead used the minimum value observed within that chemical-species group.

The *Or67d*^GAL4^ line was used to generate flies expressing *Or42a* homologs in the at1 “empty neuron” system (Kurtovic et al. 2007). We selected at1 (instead of the more commonly used antennal basiconic ab3 “empty neuron system”; Dobritsa et al. 2003) because some insects use host-derived chemicals as pheromones (Reddy and Guerrero 2004), and in *D. melanogaster* pheromones sometimes activate OSNs housed in trichoid sensilla only (Xu et al. 2005; Benton et al. 2007).

The spike amplitude differences between the two types of OSNs housed in pb3a-like and pb3b-like sensilla were less distinct in *Scaptomyza*, and therefore we could not completely rule out the possibility that we occasionally erroneously assigned spiking to each of these two OSNs types.

### Immobility Assay

To investigate the effect of AITC volatiles in wild-type *D. melanogaster* (Canton-S strain), we used a 9-cm-diameter plastic petri dish (Nunc, Denmark) with a piece of fabric mesh placed horizontally between the base and the lid, creating two chambers (supplementary fig. S1a, Supplementary Material online). The upper chamber housed 8 to 10 male flies 3 to 5 d old, and the lower chamber contained four 5 µl drops of the odor solution (or the solvent control) evenly dispersed. Because the mesh prevented the flies from reaching the bottom chamber, insects were exposed to the volatile chemicals but could not directly contact (i.e. taste) the odor solution, unless the molecules adhered to the walls of the chamber after volatilizing. After each test started, we counted the number of mobile flies every 10 min up to 1 h and calculated the percentage of mobile flies at each time point. Flies exhibiting no movement for >30 s were likely intoxicated. AITC and γ-hexalactone were diluted in either mineral oil or dimethyl sulfoxide at various concentrations, respectively. Mobility data analysis was performed using the log-rank (Mantel–Cox) test (Mantel 1966). The complete immobility assay dataset is compiled in supplementary file 3, Supplementary Material online.

### Positional Olfactory Assay

To study the olfactory orientation of insects toward odors, we conducted assays with non-starved 3 to 4 days old mated females (supplementary fig. S1b, Supplementary Material online). Flies (*n* = 10 to 12 per test) were anesthetized on ice (5 to 7 min) and placed in a small piece of clear Tygon tube, capped in both sides with a conical PCR plastic Eppendorf. After another about 4 to 5 min, the Tygon tube with the anesthetized flies was uncapped and connected to the cut ends of two glass Pasteur pipettes and the assay started; flies usually resumed activity after about 3 to 4 min. Each of the two opposite ends of the pipettes were connected to a 1.75-ml glass vial containing 10 µl of the odor solution (AITC 1: 500 vol/vol or γ-hexalactone 1:100 or 1:10 vol/vol) or 10 µl of the control solvent (mineral oil or dimethyl sulfoxide), respectively. Tests with apple cider vinegar used 30 µl instead, and water was used as a control. The distal ends of the pipettes were separated from the glass vials with a small piece of fabric mesh, which allowed the odorant to diffuse into the pipettes while also preventing insects from contacting the odor source (supplementary fig. S1b, Supplementary Material online). The odor and control sides were switched between assays. Assays were conducted on a white surface under white light at 21 to 24 °C, about 2 to 6 h after lights were on. Once each assay started, the number of flies in the pipette closest to the vial with the odor (referred to as “odor side” or odorous tube” for simplicity), in the pipette closest to the vial with the solvent control (“control” side/tube), and in the Tygon tube that connected both pipettes (release site) were counted every 5 min until 35 min, and then again at 65 min in the case of tests with AITC. For each assay, we calculated the % of insects that made a choice for one or the other tube as [(#of insects in the odor side + # of insects in the control side)/total number of insects released] × 100. The percentage of insects that choose the odorous tube was calculated based on the total number of insects that made a choice as [# of insects in the odor side/(# of insects in the odor side + # of insects in the control side)] × 100. Assays in which less than 40% of insects made a choice for either side at all time points were discarded (<5% of assays). For each fly genotype and odorant, the % of insects that choose the odor side at each time point was compared against the median value expected under the null hypothesis that insects distributed at random between the two tubes (50% of insects in each tube) using one-sample signed rank tests. Thus, we assessed whether the insects significantly avoided the odorous tube (if median < 50%), preferred it (if median > 50%), or showed a random preference (median ∼50%). In some cases, at each time point and for each odor (and concentration when applicable), the responses of the null mutants (*Or42a^−/−^*, *Or7a^−/−^*, and *Or35a^−/−^*) and their genetic background control (line 6834 listed above) tested in parallel (i.e. in the same days) were compared via Mann–Whitney *U* tests. In all cases, results were considered significant if *P* < 0.05. In most cases, we used two-tailed tests (e.g. for testing median_1_ ≠ median_2_), but in a few cases, we used one-tailed tests (e.g. for specifically testing whether median_1_ > median_2_ or whether median_1_ < median_2_; supplementary fig. S2f to i, Supplementary Material online). The positional olfactory assay data are included in supplementary file 4, Supplementary Material online. In this and all behavioral assays, we used the line # 6834 instead of the more standard *w^1118^*, because the latest have visual defects that interfere with normal behavior (Ferreiro et al. 2017).

### Consumption Assay

This behavioral assay (described in Reisenman and Scott 2019) measures if the presence of an odorant affected consumption of an appetitive solution. Groups of 2 to 4 days old mated female flies (*n* = 11 to 15) were wet-starved for 24 h and then transferred to a vial containing a piece of filter paper (2.7 cm diameter, Whatman, Cat. No 1001 125) impregnated with 160 µl of 50 mM D-glucose (Sigma-Aldrich, USA) dyed blue with Erioglaucine (0.25 mg/ml, Sigma-Aldrich, St. Louis, Missouri, USA; supplementary fig. S1c, Supplementary Material online). Flies were allowed to feed for 15 min (10 min in tests with γ-hexalactone), frozen (>60 min), and the amount of blue dye in the flies' abdomen was scored blind to treatment (see below). The odor source consisted of a strip of filter paper (0.25 cm wide × 1.5 cm long) impregnated with either 10 µl of an odorant solution (test) or 10 µl of the solvent (control), which was placed inside a container (1.3 cm long × 0.75 cm diameter) with a meshed bottom affixed to the vial's flug (supplementary fig. S1c, Supplementary Material online). This allowed diffusion of odors into the fly vial but prevented flies from contacting the odor source. Control tests, with vials containing food solution but only the solvent control inside the meshed container, were conducted in parallel with experimental tests to control for fly cohort and day-to-day variability.

Food consumption was estimated by scoring individual flies in each vial blind to treatment using the following 5-point scale (Reisenman and Scott 2019): 0 (no dye = no food), 0.25 (“trace” of blue dye), 0.5 (up to one-fourth of the abdomen dyed blue), 1 (more than one-fourth but less than half of the abdomen dyed blue), and 2 (more than half of the abdomen dyed blue). For each vial, a single feeding score value was calculated as [(0 × *n*_0_ + 0.25 × *n*_0.25_ + 0.5 × *n*_0.5_ + 1 × *n*_1_ + 2 × *n*_2)_/*N*], where *n*_(0 to 2)_ denotes the number of flies in each score category and *N* the total number of flies/vial. Feeding scores from each test vial (flies offered food in presence of an odor) were normalized to the averaged feeding score of control vials (flies of the same genotype offered food in absence of odor) assayed on the same day. Normalized feeding scores for each genotype and odor were compared against the null hypothesis (median feeding score = 1) using one-sample signed rank tests. That is, medians not different from 1 indicate that the odorant did not reduce neither enhanced consumption, while medians significantly less than 1 or more than 1 indicate feeding aversion and enhancement, respectively. Normalized data from control and mutant flies were compared using Mann–Whitney *U* tests. In all cases, results were considered statistically significant if *P* < 0.05. The consumption assay data are compiled in supplementary file 5, Supplementary Material online.

### RNA-Sequencing of Maxillary Palps

Newly emerged adults of *S. flava* and *S. pallida* were collected from our colony and kept in humidified vials with 10% honey water until dissection, to minimize potential differences in nutrition resulting from differences in the two species' larval diet. Flies that were 3 to 10 days old flies were anesthetized with CO_2_, and their maxillary palps were hand-dissected using forceps. Approximately 100 to 120 flies were pooled for a single sample. The dissected tissues were directly collected in LB + TGA lysis buffer from ReliaPrep RNA Tissue Miniprep System (Promega, USA) and homogenized using a Biomasher Standard homogenizer (Takara Bio Inc., USA) in a dry ice ethanol bath. The sample lysates were stored at −80 °C until RNA extraction. Total RNAs were extracted from the lysates using ReliaPrep RNA Tissue Miniprep System (Promega, USA) according to the manufacturer's protocol, and quantified using a Qubit RNA High Sensitivity kit (Thermo Fisher Scientific, USA). Library preparation was performed at the Functional Genomics Laboratory at UC Berkeley. Due to the low yields of our maxillary palp-derived total RNAs, complementary DNA (cDNA) libraries were first produced by Takara SMART-Seq mRNA Ultra-low input RNA kit (Takara Bio Inc., USA) with eight cycles of PCR for the amplification, and then processed by KAPA HyperPrep kit for DNA (Roche Sequencing, USA) with nine cycles of PCR for attaching in-house sequencing adapters and index primers. cDNA libraries were then sequenced on an Illumina NovaSeq 6000 150 PE S4 flowcell, targeting 25 M read pairs per library by the UC Berkeley Vincent J. Coates Genomics Sequencing Laboratory. For read mapping, we used previously reported reference genome assemblies and gene annotations from *S. flava* (Peláez et al. 2023) and *S. pallida* (Kim et al. 2021) for subsequent bioinformatic analyses. Raw RNA-sequencing (RNA-seq) reads were filtered using Fastp v0.21.0 (Chen et al. 2018) and mapped to the respective reference genomes using STAR v2.7.1a (Dobin et al. 2013) to generate multiple alignment (BAM) files, which were then converted to read count data using HTseq v0.9.1 (Anders et al. 2015). Count data for the *Or* gene family were converted to reads per million (RPM; supplementary file 6, Supplementary Material online).

### Hybridization Chain Reaction RNA FISH

One to four days old female *S. flava* and *S. pallida* were collected and anesthetized with CO_2_. Whole mouthparts were removed and immediately placed in 2 ml of fixative (4% vol/vol paraformaldehyde in 1× phosphate buffer saline with 3% vol/vol Triton X-100 added, PBST (1xPBS + 0.1% Tween 20)) in LoBind Eppendorf tubes, and fixed for 22 h at 4 °C on a nutator. For Hybridization chain reaction RNA FISH, we followed the manufacturer's instruction (Molecular Instruments, Inc., Los Angeles, California, USA).

Samples were stained with 300 nM DAPI (4′,6-diamidino-2-phenylindole) in 0.1% PBST for 15 min and then washed thrice with 0.1% PBST for 5 min. Tissues were transferred to a microscope slide and mounted in a drop of ProLong diamond antifade mounting (Life Technologies Corp., Eugene, Oregon, USA) and stored at 4 °C until examination. Confocal imaging of fixed samples was performed using a Zeiss LSM 880 microscope in the AiryScan mode. Raw images were processed using Zeiss ZEN Black software. Orco-positive cells (visualized with the 488 nm laser) and Or42a-positive cells (visualized with the 633 nm laser) were manually counted using the “Cell Counter” plugin in Fiji (ImageJ) software. supplementary file 7, Supplementary Material online, contains cell counts from RNA FISH experiments.

### *Scaptomyza Or42a* Gene Cloning and Generation of *UAS* Lines

RNA was isolated from 15 to 25 days old laboratory-reared adults of both sexes of *S. pallida* and *S. flava* using the ReliaPrep Miniprep system (Promega, Wisconsin, USA). cDNA was synthesized using the qScript cDNA Supermix (Quantabio, Beverly, Massachusetts, USA). The paralogs were amplified via touchdown PCR, using primers that target the highly variable regions of the 5′ and 3′ untranslated regions (UTRs). The single *Or42a2* gene of *S. pallida* was amplified using touchdown PCR with primers targeting the coding region (Q5 DNA Polymerase, #M0491, NEB) (supplementary file 8, Supplementary Material online). All PCR products underwent gel purification (11-300C, Zymo Research) and were subsequently cloned using the Gibson Assembly (E2611S, NEB) following the manufacturer's instructions into the UAST-attB vector (DGRC Stock 1419; RRID:DGRC1419, Bischof et al. 2007). The ligation products were transformed into DH5α competent cells (T3007, Zymo Research). After confirming the sequences using Sanger sequencing, the 5′ and 3′ UTRs of the plasmids containing the *S. flava Or42a* paralogs were removed. This was achieved by first amplifying the coding region using PCR, followed by gel purification and ligation back into UAST-attB vector via Gibson Assembly. The resultant plasmids were verified using Sanger sequencing. Finally, these plasmids were microinjected into the attP40 site (*P*{nos-phiC31\int.NLS}X, *P*{CaryP}attP40, line # 25709, Rainbowgene) to create transgenic lines for *S. pallida Or42a*, *S. flava Or42a2*, *S. flava Or42a3*, and *S. flava Or42a4*. All lines were confirmed by Sanger sequencing prior to experiments.

### Screening of Candidate Amino Acids Using AlphaFold2 3D Structural Prediction

CDS (Coding DNA sequence) of *S. flava Or42a3* and *S. flava Or42a4* were confirmed by palp RNA-seq data using IGV_2.16.0. CDSs of *S. flava Or42a3* and *S. flava Or42a4* were then used as inputs into ColabFold (Jumper et al. 2021; Mirdita et al. 2022). Then the output models ranked first (rank1) were selected, visualized, and 3D aligned by using PyMol 2.5.3. We focused on the regions where predicted local distance difference test scores exceed 70, as structures with lower values are often unreliable (Mariani et al. 2013). We focused on the S5-S6 transmembrane helices because previous studies suggested that the binding pockets of odorant receptors are located in the transmembrane region (Butterwick et al. 2018; Del Mármol et al. 2021; Zhao et al. 2024), and our amino acid alignment of *S. flava* Or42a3 and *S. flava* Or42a4 provided the highest root mean square deviation scores in this region. In silico substitutions of amino acids were performed individually on *S. flava* Or42a3, and the resulting sequences were then re-input into ColabFold, the model with rank1 was selected, and the structures were 3D aligned with that of *S. flava* Or42a4. This process of in silico mutation and alignment was repeated until the root mean square deviation scores for the S5-S6 region were reduced to a value comparable to the other regions (∼0.1 Å). All pdb files used in this study are included in supplementary file 9, Supplementary Material online.

### Site-Directed Mutagenesis

Conventional PCR was conducted using plasmids of *S. flava Or42a3* as backbone (Q5 DNA polymerase, #M0491, NEB; supplementary file S1, Supplementary Material online). Primers were designed to introduce the point mutations (supplementary file 1, Supplementary Material online). The PCR product underwent gel purification (11-300C, Zymo Research) and the methylated plasmids were digested with DpnI for 1 h at 37 °C (QuickChange, Agilent Technology, USA). Ligation was performed by incubation at 16 °C for 30 min (DNA ligation kit Mighty mix, Takara, Japan) and the products were transformed into DH5α competent cells (Takara, Japan). After confirming the sequence using Sanger sequencing, the plasmid was microinjected into the attP40 site (P{nos-phiC31\int.NLS}X, P{CaryP}attP40, line # 25709, Rainbowgene) to create the mutant A181D S307P fly line. The mutations were confirmed by sequencing prior to experiments.

## Supplementary Material

msaf164_Supplementary_Data

## Data Availability

The data presented in this study are available on request from the corresponding authors.
