# Supplementary material for: Odorant Receptors Mediating Avoidance of Toxic Mustard Oils in Drosophila melanogaster Are Expanded in Herbivorous Relatives
Source: Mol Biol Evol. 2025 Jul 4;42(9):msaf164. doi: 10.1093/molbev/msaf164 (PMC12448936; doi:10.1093/molbev/msaf164)

Supp. Figure 1

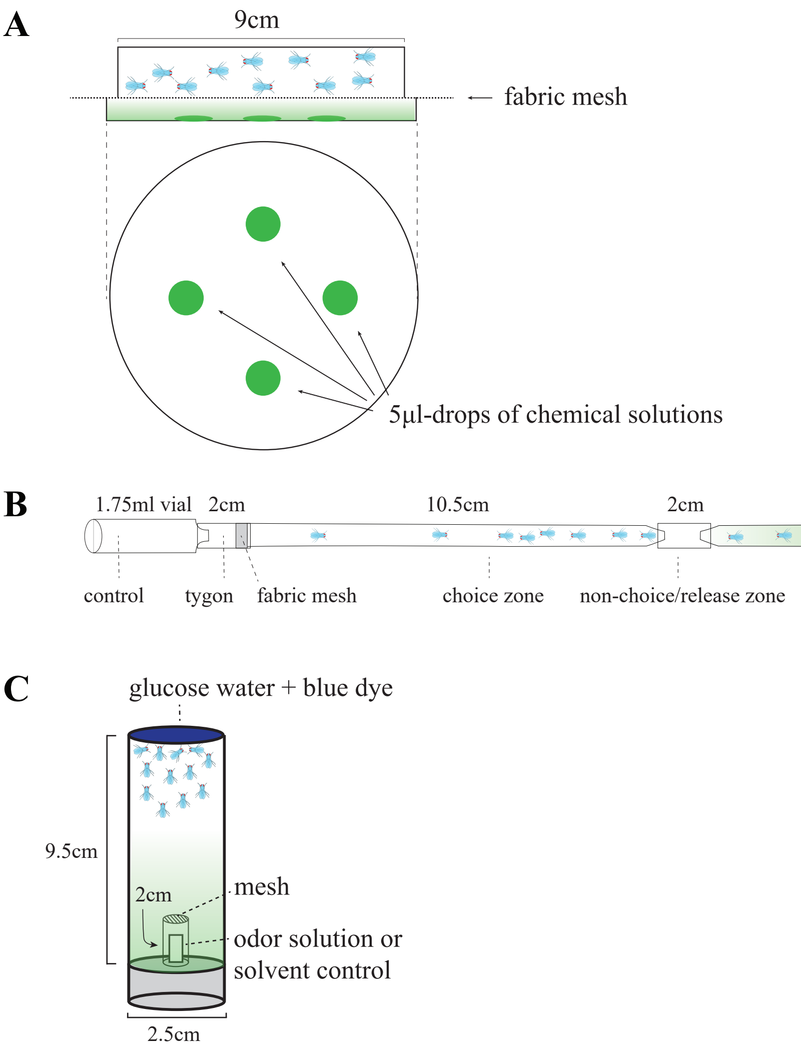

Supp. Figure 2

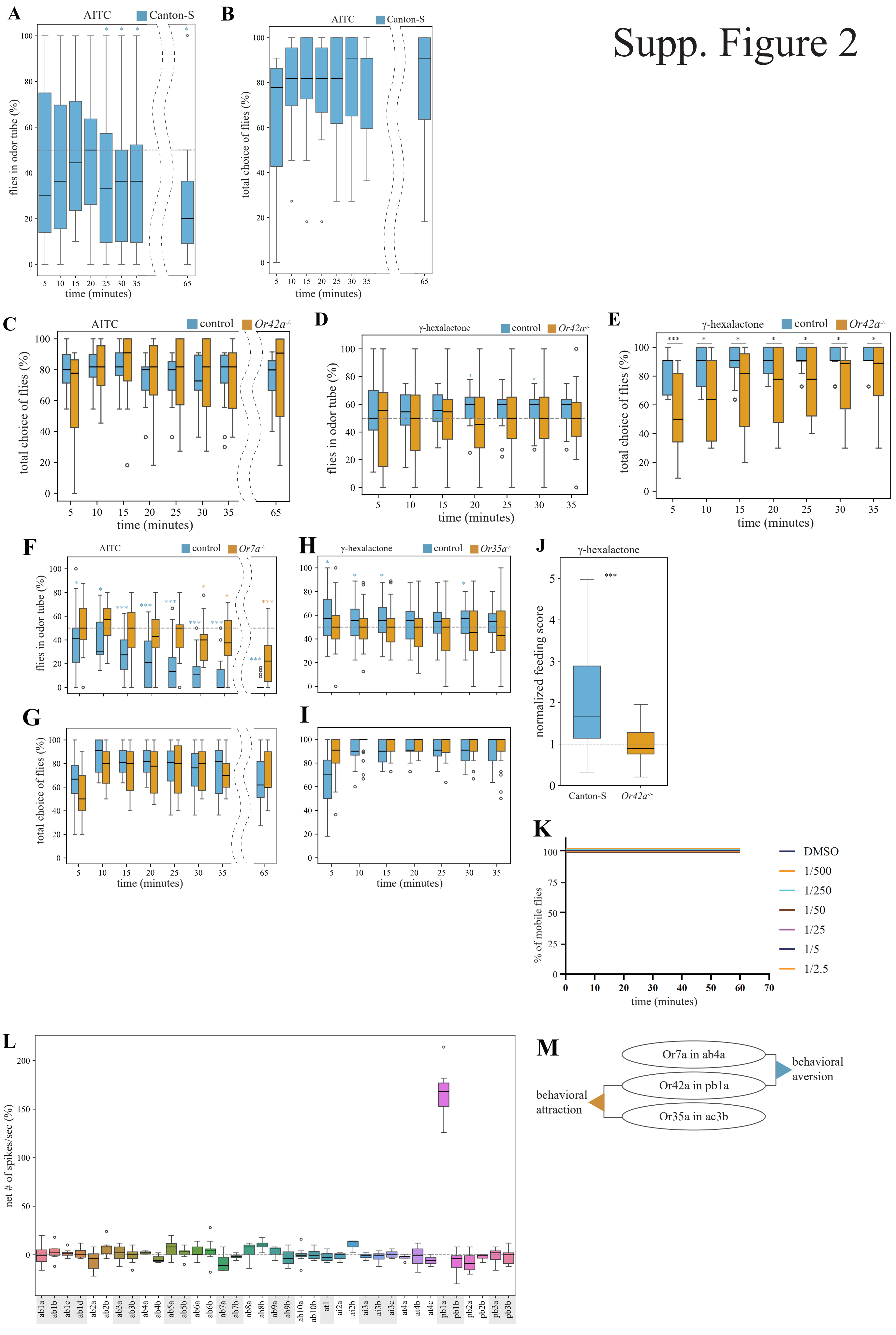

Supp. Figure 3

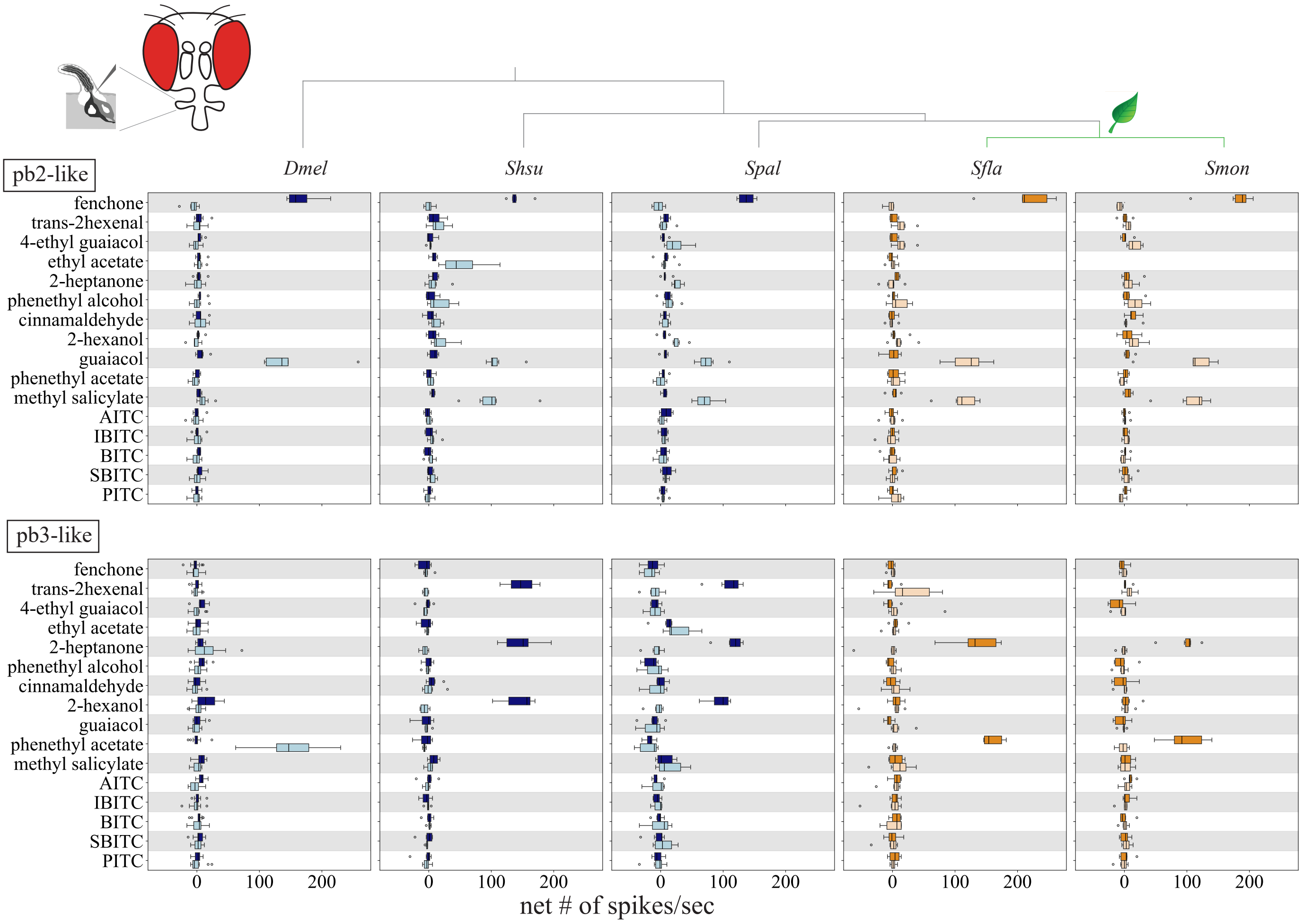

Supp.Figure 4

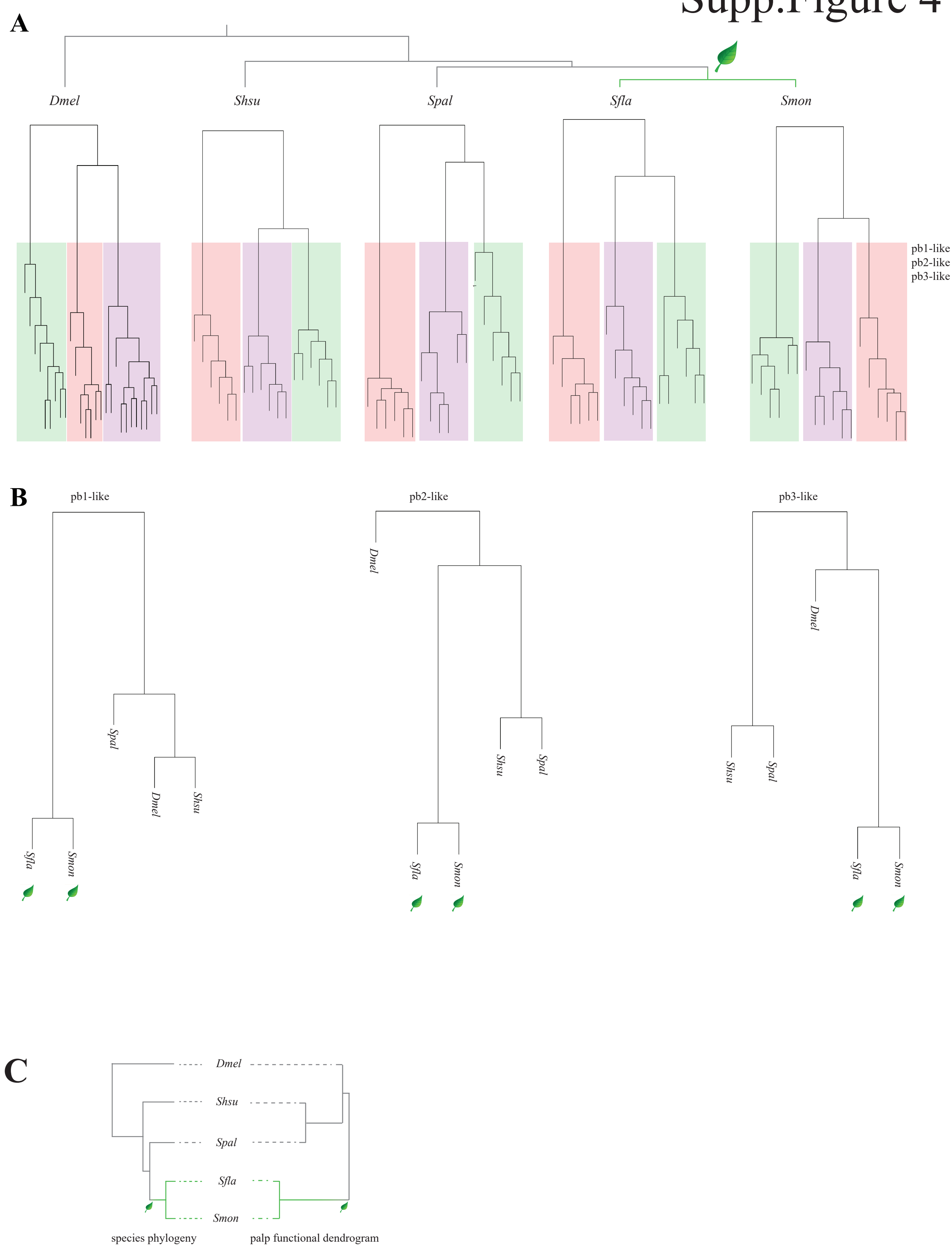

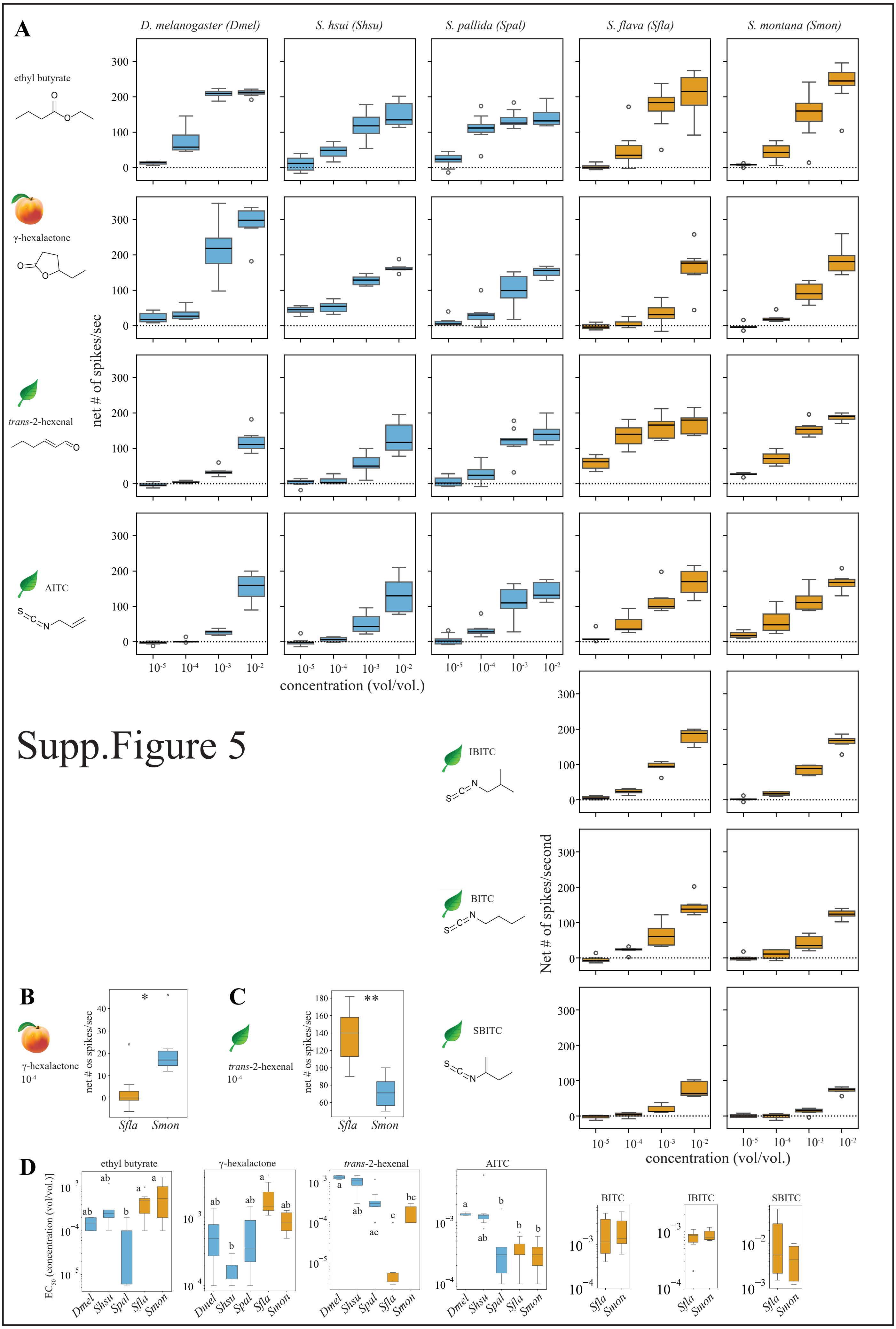

Supp.Figure 5

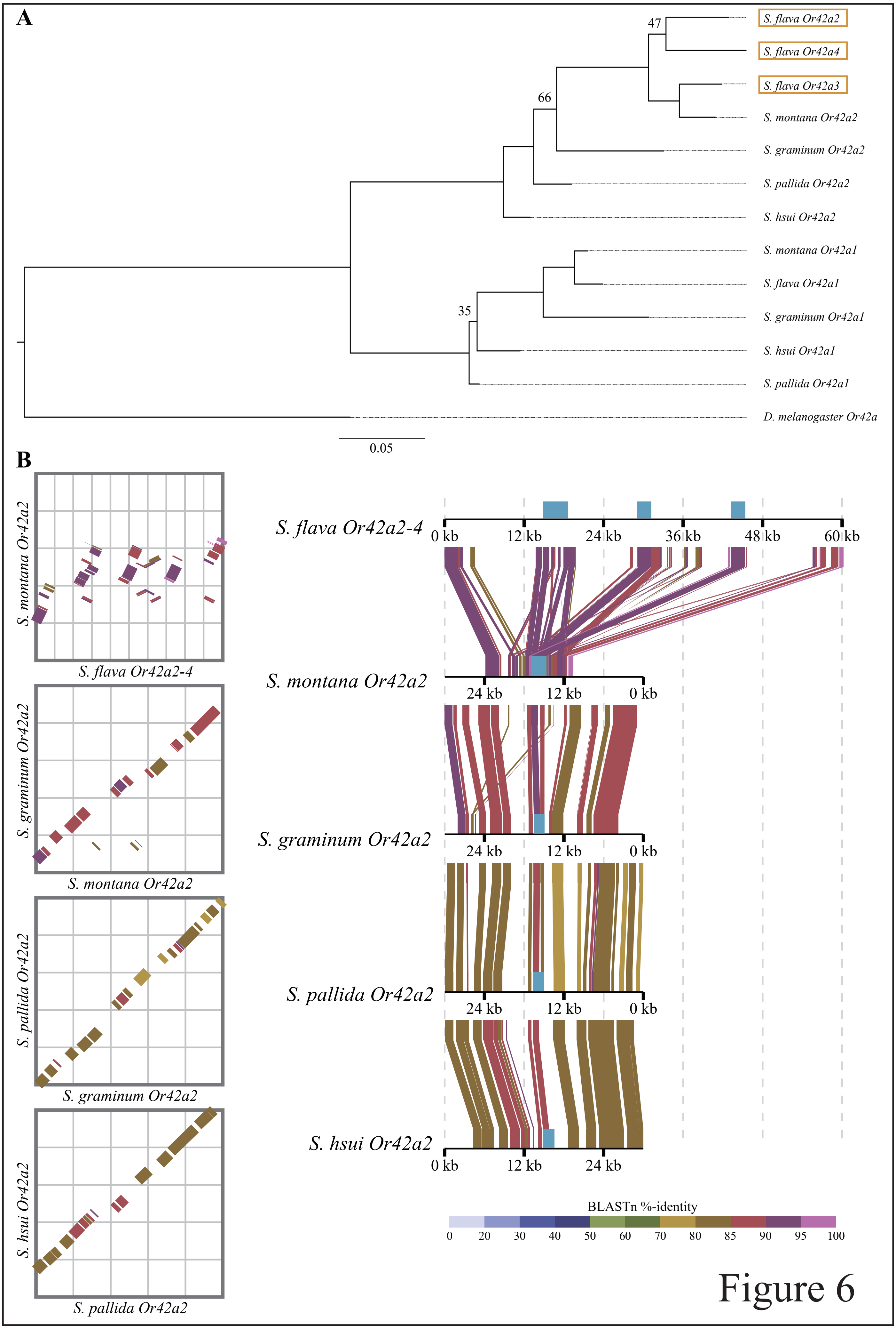

A
47
66
35
S. flava Or42a2
S. flava Or42a4
S. flava Or42a3
S. montana Or42a2
S. graminum Or42a2
S. pallida Or42a2
S. hsui Or42a2
S. montana Or42a1
S. flava Or42a1
S. graminum Or42a1
S. hsui Or42a1
S. pallida Or42a1
D. melanogaster Or42a
0.05
B
S. montana Or42a2
S. flava Or42a2-4
S. graminum Or42a2
S. montana Or42a2
S. pallida Or42a2
S. graminum Or42a2
S. hsui Or42a2
S. pallida Or42a2
S. flava Or42a2-4
0 kb
12 kb
24 kb
36 kb
48 kb
60 kb
S. montana Or42a2
24 kb
12 kb
0 kb
S. graminum Or42a2
24 kb
12 kb
0 kb
S. pallida Or42a2
24 kb
12 kb
0 kb
S. hsui Or42a2
0 kb
12 kb
24 kb
BLASTn %-identity
0 20 30 40 50 60 70 80 85 90 95 100

Figure 6

Supp. Figure 7

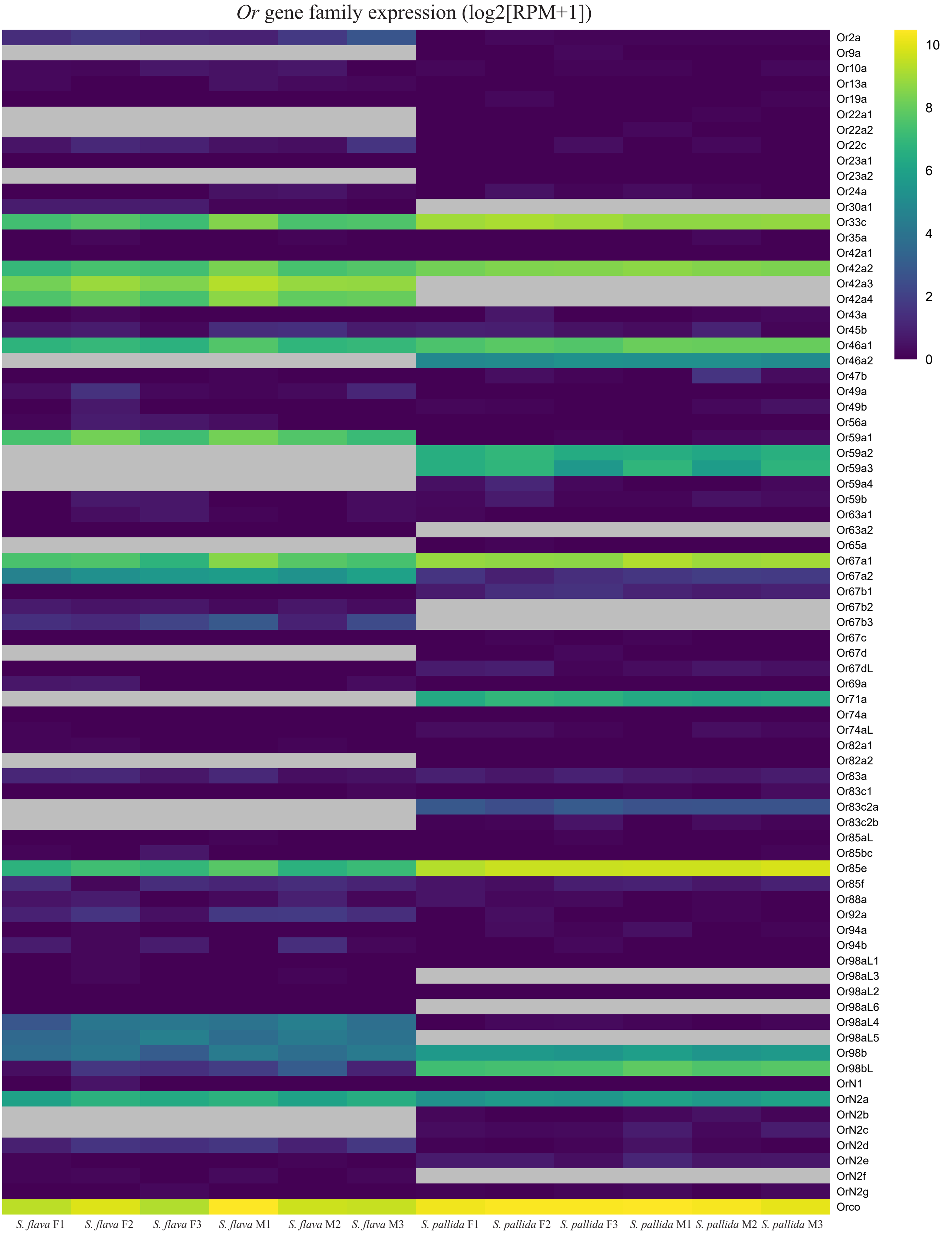

Supp. Figure 8

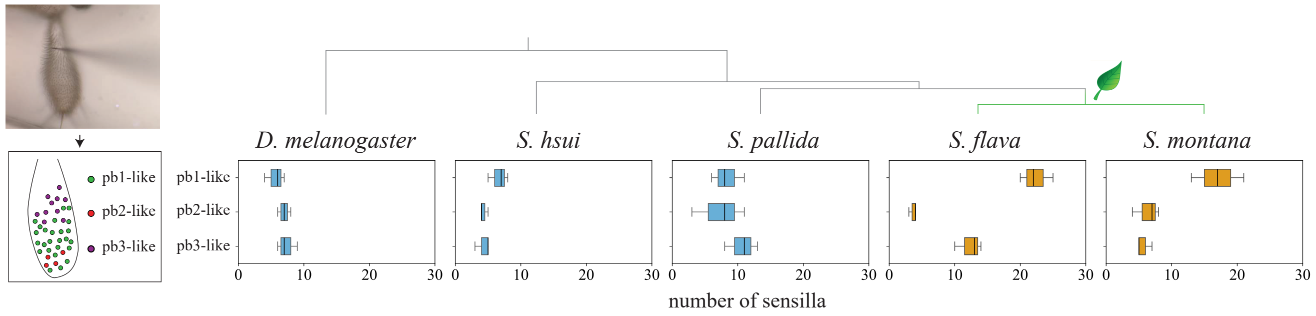

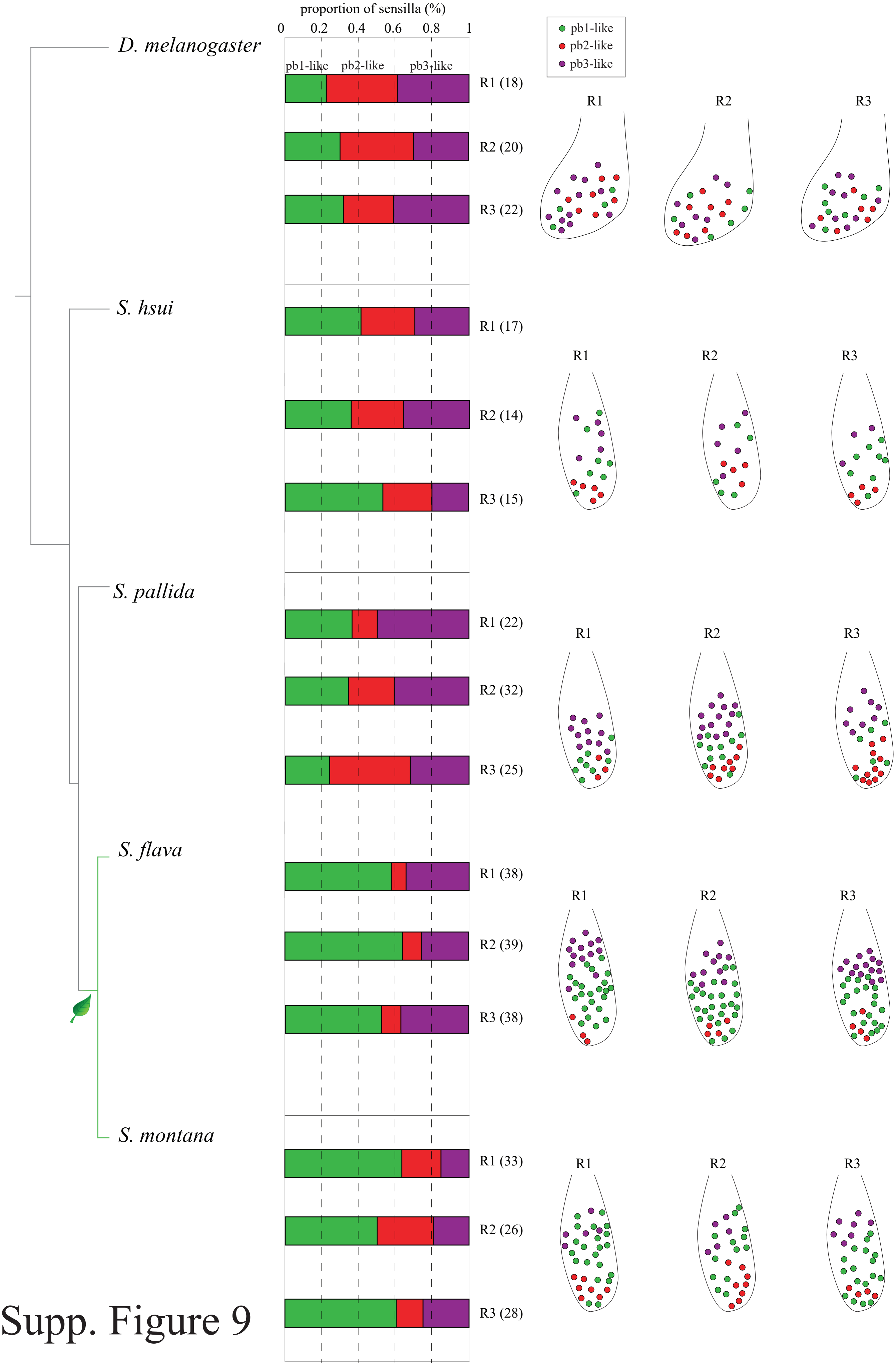

Supp. Figure 9

Supp. Figure 10

A

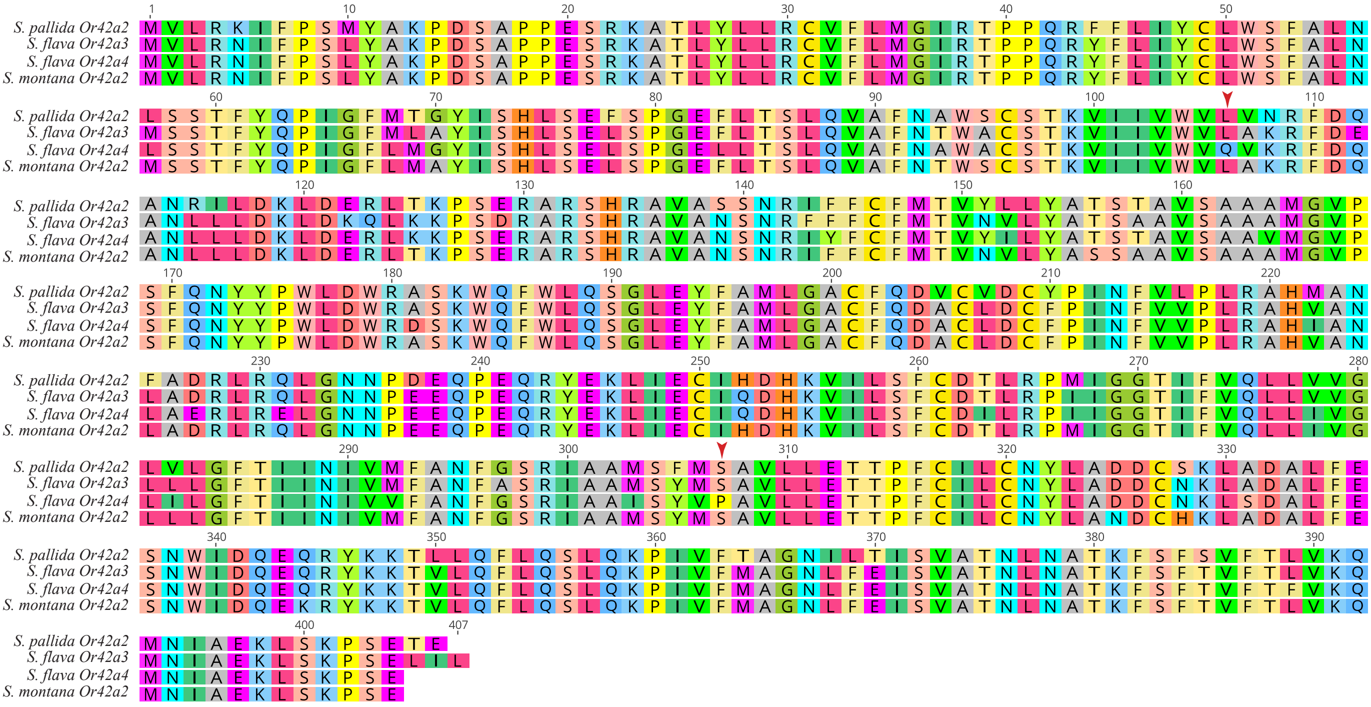

B

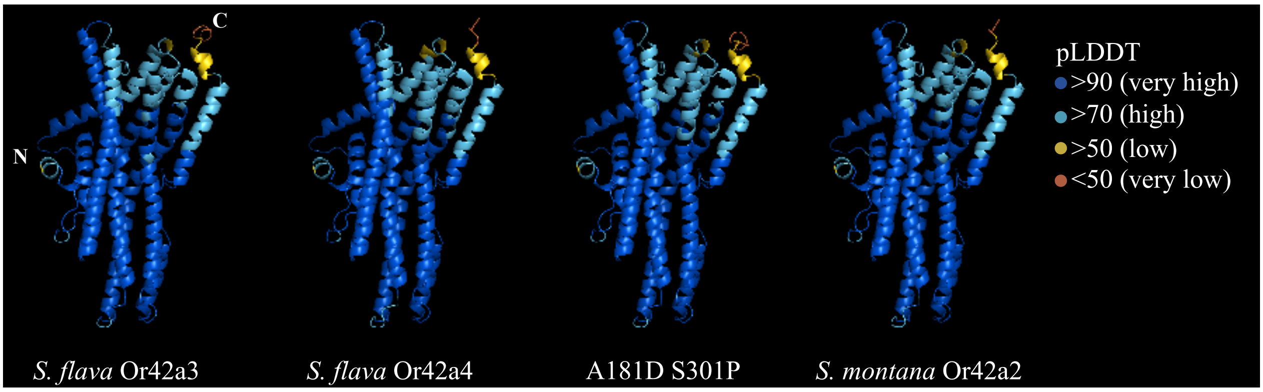

Supp. Figure 11

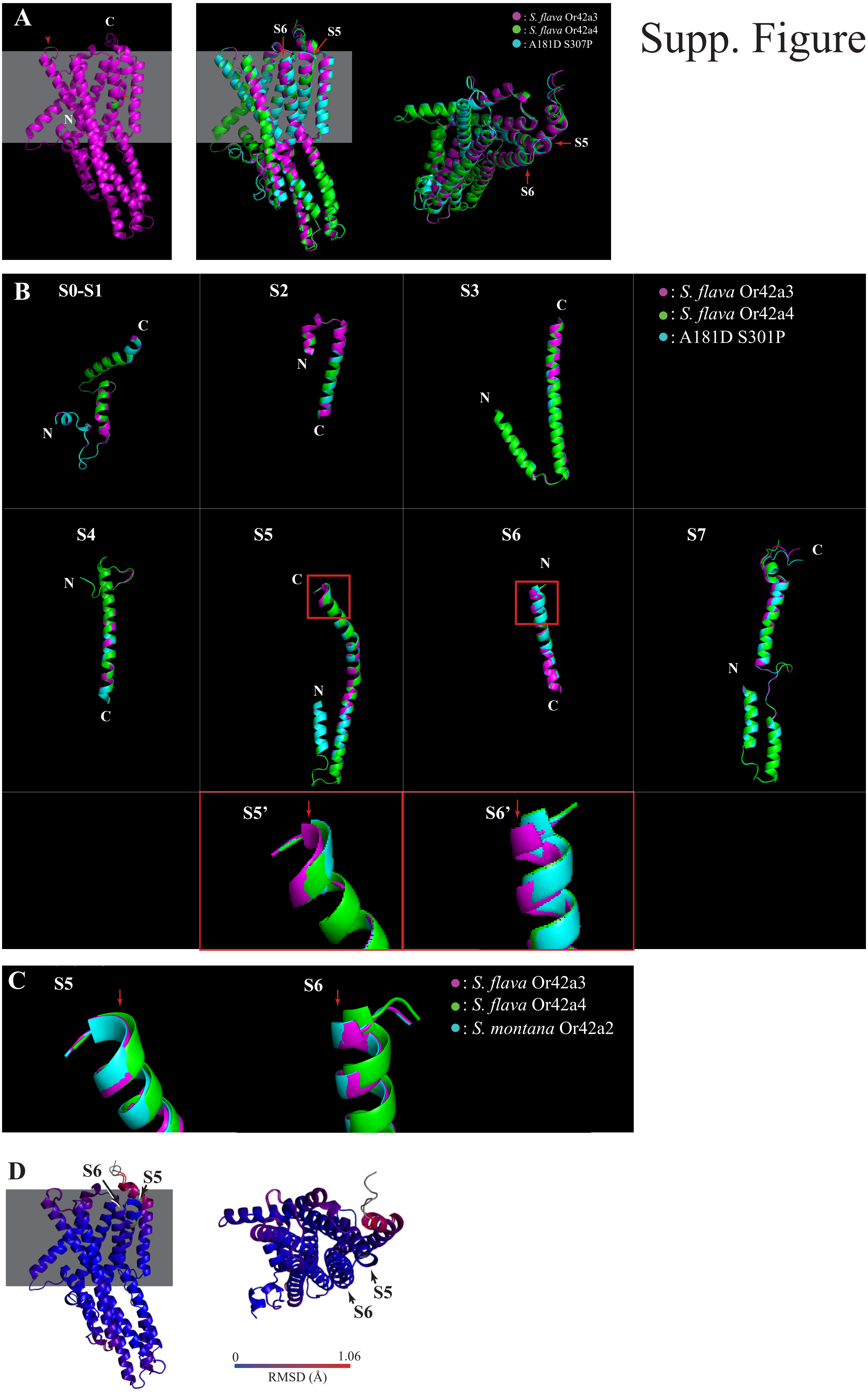

Supp. Figure 12

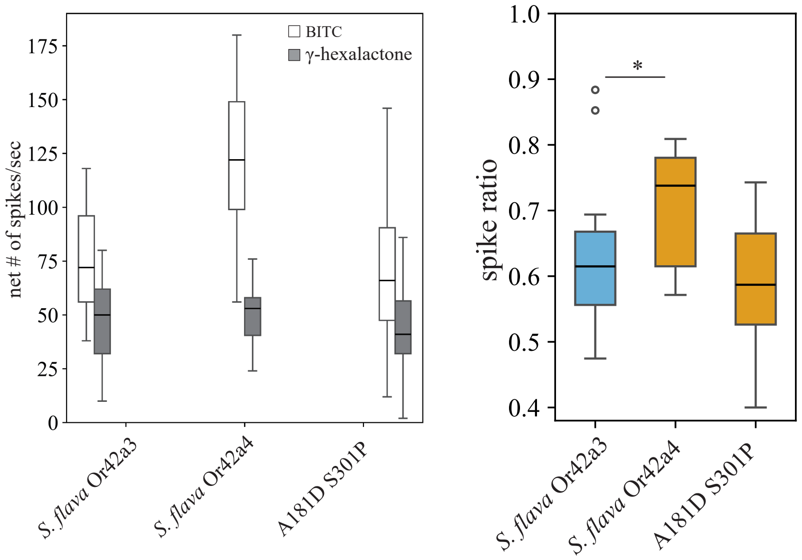

Supp. Figure 13

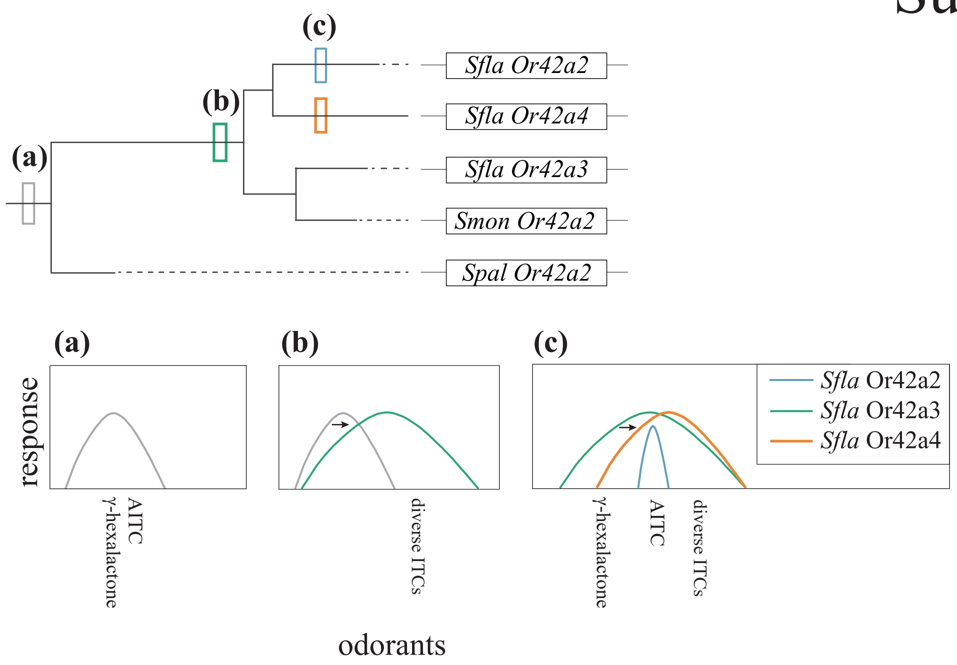

Supplement: msaf164_Supplementary_Data [file msaf164_supplementary_data.zip › Or42a_Supplement_Fig_final250716.pdf]
